# The Expression of TRIM6 Activates the mTORC1 Pathway by Regulating the Ubiquitination of TSC1-TSC2 to Promote Renal Fibrosis

**DOI:** 10.3389/fcell.2020.616747

**Published:** 2021-02-09

**Authors:** Weiwei Liu, Yang Yi, Chuanfu Zhang, Baojuan Zhou, Lin Liao, Wenrui Liu, Jing Hu, Qiming Xu, Jie Chen, Jianrao Lu

**Affiliations:** ^1^Department of Nephrology, Seventh People's Hospital of Shanghai University of Traditional Chinese Medicine, Shanghai, China; ^2^Department of Nephrology, Jing'an District Central Hospital of Shanghai/ Jing'an Branch, Huashan Hospital, Fudan University, Shanghai, China

**Keywords:** TRIM6, angiotensin II, renal fibrosis, mTOR, TSC1, TSC2

## Abstract

Renal fibrosis is considered as the final pathway of all types of kidney diseases, which can lead to the progressive loss of kidney functions and eventually renal failure. The mechanisms behind are diversified, in which the mammalian target of rapamycin (mTOR) pathway is one of the most important regulatory pathways that accounts for the disease. Several processes that are regulated by the mTOR pathway, such as autophagy, epithelial-mesenchymal transition (EMT), and endoplasmic reticulum (ER) stress, are tightly associated with renal fibrosis. In this study, we have reported that the expression of tripartite motif-containing (TRIM) protein 6, a member of TRIM family protein, was highly expressed in renal fibrosis patients and positively correlated with the severity of renal fibrosis. In our established *in vitro* and *in vivo* renal fibrosis models, its expression was upregulated by the Angiotensin II-induced nuclear translocation of nuclear factor-κB (NF-κB) p50 and p65. In HK2 cells, the expression of TRIM6 promoted the ubiquitination of tuberous sclerosis proteins (TSC) 1 and 2, two negative regulators of the mTORC1 pathway. Moreover, the knockdown of TRIM6 was found efficient for alleviating renal fibrosis and inhibiting the downstream processes of EMT and ER in both HK2 cells and 5/6-nephrectomized rats. Clinically, the level of TRIM6, TSC1/2, and NF-κB p50 was found closely related to renal fibrosis. As a result, we have presented the first study on the role of TRIM6 in the mTORC1 pathway in renal fibrosis models and our findings suggested that TRIM6 may be a potential target for the treatment of renal fibrosis.

## Introduction

Kidney diseases, such as chronic kidney diseases (CKD), glomerulonephritis, and polycystic kidney disease are usually caused by injuries and damages that influence normal renal functions. Associated with other common diseases, e.g., diabetes, hypertension, and pathogenic infections, kidney diseases have currently become more threatening to human life, affecting over 25 million American adults (Coresh et al., 2007). Through influencing the vasculature, glomerulus, and tubulointerstitium of the kidney, various factors, including immunological, mechanical, metabolic, and toxic factors, attribute to kidney diseases, leading to end-stage renal failure (Kato et al., 2008; Arnold et al., 2016). Renal fibrosis, occurring in the parenchyma, is considered as the final pathway for all types of chronic and progressive nephropathies (Humphreys, 2018).

Epithelial-mesenchymal transition (EMT) is a reversible cellular process in which epithelial cells transform into a transient quasi-mesenchymal cell states (Dongre and Weinberg, 2019). During this process, epithelial cells progressively lose the epithelial morphology and further adopt a spindle-shaped, mesenchymal morphology. EMT involves tumor progression, embryological development, wound healing, which is identified as one of the driving forces for fibrosis (Lamouille et al., 2014). As an upstream EMT-regulating process, endoplasmic reticulum (ER) stress is responsible for the regulation of calcium storage, lipid biosynthesis, and protein folding and protein transport (Ulianich et al., 2008; Tanjore et al., 2011; Zhou et al., 2020). The mammalian target of rapamycin (mTOR) pathway plays a central role in regulating cell growth, proliferation, ER stress, metabolism, and autophagy. It exhibits indispensable functions in regulating renal cell homeostasis and cellular processes, which is critical to kidney diseases (Lui et al., 2006; Mostov, 2006; Lieberthal and Levine, 2009; Grahammer et al., 2014; Inoki, 2014; Fantus et al., 2016; Yao and Inoki, 2016). In the mTOR pathway, the mTORC1 signaling is mainly responsible for regulating these processes (Yuan et al., 2013; Rabanal-Ruiz et al., 2017). The upstream mTORC1 regulators TSC1 and TSC2 form a heterodimeric complex, which negatively regulates mTORC1 through activating GTPase Rheb, thereby affecting the activities of downstream effectors and cellular processes (Dibble and Cantley, 2015).

The TRIM protein superfamily consists of 70 protein members that are responsible for regulating critical cellular processes, including intracellular signaling, innate immunity, transcription, autophagy, and carcinogenesis (Hatakeyama, 2017). Several members of the TRIM protein have been implicated in renal fibrosis-related pathways (Liu, 2011; Nogueira et al., 2017), such as TGF-β1/Smads (Lee, 2018), PDGF (Wang et al., 2020), and Wnt/β-catenin (Yang et al., 2017). TRIM6 belongs to the PY/SPRY-domain-containing group. It mediates the IKKe-dependent signaling by interacting with IKKe *via* its SPRY domain (Rajsbaum et al., 2014). It also assists in the synthesis of unanchored K48-linked polyubiquitin chains to upregulate the activity of IKKe (Rajsbaum et al., 2014; Bharaj et al., 2016). However, the precise functions of TRIM6 in the TRIMosome-involved regulation are unknown. TRIM6 expression was increased in the peripheral blood samples of idiopathic pulmonary fibrosis (Li et al., 2020), but its role in fibrosis-related diseases, i.e., renal fibrosis, still remains veiled.

Angiotensin (Ang) II can not only induce EMT (Seccia et al., 2019), ER stress (Menikdiwela et al., 2019), and renal fibrosis (Mezzano et al., 2001; Xu Z. et al., 2017), but also promote the production of reactive oxygen species (ROS) that further mediates the regulation of the nuclear factor-κB (NF-κB) pathway (Morgan and Liu, 2011), a crucial process tightly associated with different renal diseases (Zhang and Sun, 2015). In this study, we have reported that TRIM6 is tightly associated with renal fibrosis by participating in the regulation of the mTORC1 signaling. When treated with Ang II, HK2 cells showed a high expression of TRIM6, which was upregulated by the Ang II-induced nuclear translocation of nuclear factor-κBp50 and p65. The expression of TRIM6 promoted the ubiquitination of TSC1 and TSC2, thereby activating mTORC1 and downstream processes of ER stress and EMT in renal fibrosis models. The knockdown of TRIM6 in HK2 cells and 5/6-nephrectomized rats remarkably improved the status of renal fibrosis, which inspires the development of TRIM6 inhibitors for treating renal fibrosis in kidney diseases.

## Materials and Methods

### Bioinformatics Analysis

The gene expression data were obtained from the GEO dataset (Access id: GSE7392, http://www.ncbi.nlm.nih.gov) (Park and Stegall, 2007). Our GSEA of pathways and genes was performed based on the GEO dataset (GSE7392) using the GSEA version 2.0 from the Broad Institute at MIT. In our analysis, the gene sets of fewer than 10 genes were excluded. Using a permutation test 1,000 times, the cutoff of *p*-values was chosen as 0.01 for the most significant pathways related to TRIM6 expression.

### Kidney Tissue Samples

In total, kidney tissue samples were obtained from 75 patients, and processed using hematoxylin–eosin staining. Renal fibrosis severity was determined on the basis of the Banff criteria (Solez et al., 2008). The 75 cases were divided into four groups (Interstitial Fibrosis/Tubular Atrophy [IF/TA] 0, ≤ 5% interstitial fibrosis of cortical area, *n* = 12; IF/TA 1, 6–25%, *n* = 22; IF/TA 2, 26–50%, *n* = 27; IF/TA 3, >50%, *n* = 14). All the protocols conformed to the ethical guidelines of the 1975 Helsinki Declaration. The present study was approved by the ethics committee of Seventh People's Hospital of Shanghai University of Traditional Chinese Medicine (Shanghai, China, Approval number: 2019-AR-005). Written consent was obtained from all patients.

### Cell Cultures and Angiotensin II Treatment

Human kidney-2 (HK2) cells, which are immortalized human renal proximal tubular epithelial cells, were obtained from the cell bank of Shanghai Biology Institute, Chinese Academy of Science (Shanghai, China). The cells were cultured in the RPMI-1640 medium (Hyclone, Logan, UT, USA) supplemented with 10% fetal bovine serum (Invitrogen) and 1% streptomycin/penicillin (Invitrogen). Cells were incubated at 37°C, with 5% CO2. At 60–70% of confluence, cells were growth-arrested in a serum-free medium for 24 h before the experiments. HK2 cells were treated with 0, 0.25, 0. 5, 1.0, 2.0, and 4.0 μM of angiotensin II (Ang II). Cell morphology was observed under the Olympus inverted microscope (Lake Success, NY, USA).

### RNA Isolation and Quantitative RT-PCR

Total RNA was extracted using TRIzol reagent (Invitrogen) according to the manufacturer's instructions. The mRNA levels of indicated genes were determined by quantitative RT-PCR using the SYBR®Green (Thermo Fisher Scientific) on an ABI 7300 instrument (Applied Biosystems), with *GAPDH* being used as an internal control. All reactions were conducted using the following cycling parameters: 95°C for 10 min, followed by 40 cycles of 95°C for 15 s and 60°C for 45 s. The verification of specific product amplification was performed by dissociation curve analysis. The comparative Ct method was used for the quantification of the transcripts. The fold-change for target genes normalized by internal control was determined by the formula 2^−ΔΔCT^. All data represent the average of three replicates. The primers are listed in Supplementary Table 1.

### Preparation of Total Cell Lysates, Cytosolic Fraction and Nuclear Extracts, and Western Blot Analysis

Total cell lysates were prepared with a radioimmunoprecipitation buffer containing the proteinase inhibitor (Beyotime). The cytosolic fraction and nuclear extracts were prepared with NE-PER™ Nuclear and Cytoplasmic Extraction Reagents (Thermo Fisher Scientific) as per the manufacturers' instructions. Proteins were separated by sodium dodecyl sulfate-polyacrylamide gel electrophoresis (SDS-PAGE) and electroblotted onto nitrocellulose membranes (Millipore, Billerica, WI, USA). After blocking with 5% skimmed milk, the membranes were incubated overnight at 4°C with primary antibodies (Supplementary Table 2) following the instructions. After washing away the unbound antibody, the membranes were incubated at room temperature for 1 h with the HRP-conjugated rabbit secondary antibody (Beyotime, Shanghai, China). The enhanced chemiluminescence system (ECL) (Millipore) was employed to visualize the protein bands. Densitometry analysis was performed using Imag J software (http://rsb.info.nih.gov/ij/, Bethesda, MD, USA).

### Lentivirus Preparation

Short hairpin RNA (shRNA) oligos targeting TRIM6 (Supplementary Table 3) were annealed and cloned into *Age*I- and *Eco*RI-digested pLKO.1 (Addgene, Cambridge, MA, USA). The full-length coding sequences of human *TRIM6, TSC1*, and *TSC2* were cloned into pLVX-puro (Clontech, Palo Alto, CA, USA). The lentivirus was produced in HEK293T cells along with the packaging plasmids of psPAX2, and pMD2.G.

### Small Interference RNA (siRNA)

siRNAs targeting NF-κB p65 (sip65#1, #2, #3) and NF-κB p50 (sip50#1, #2, #3) and scrambled siRNA (siNC) were synthesized by Genepharma (Shanghai, China) (Supplementary Table 4). To knock down the expression of NF-κB p65 and NF-κB p50, HK2 cells were transfected with siRNAs by Lipofectamine 2000 (Invitrogen, Carlsbad, CA, USA) according to the manufacture's protocol.

### Transwell Assay

Transwell assay was performed using chamber with 8 μm pore filters (Corning, New York, NY, USA). HK2 cells were transduced with virus expressing TRIM6 shRNAs (shTRIM6-1#, 2#) or the control shRNA (shNC). After 24 h, the cells were trypsinized and plated onto the upper chamber. 1 μM of Ang II was added to the lower chamber. After incubation for 24 h at 37°C, non-migrating cells were completely removed, and the migrated cells were fixed in 4% paraformaldehyde, stained with 0.5% crystal violet, visualized under a microscope, and counted in five random fields.

### Evaluation of ROS Production by Flow Cytometry

Cells were harvested, washed with ice-cold PBS, and probed with 10 μM 2′,7′-Dichlorodihydrofluorescein diacetate (DCFH-DA) (Beyotime) at 37°C for 20 min. At the end of incubation, the samples were analyzed with flow cytometry (BD Biosciences, Franklin Lakes, NJ, USA). The experiment was performed using an excitation wavelength of 480 nm and an emission wavelength of 525 nm.

### Immunoprecipitation (IP) and Liquid Chromatography/Mass Spectrometry (LC/MS) Analysis

pCMV-Tag2-TIRM6 or pCMV-Tag2 vector was transfected into HEK293T cells, which were harvested and extracted in the RIPA buffer 48 h later. The overexpression of FLAG-TRIM6 was confirmed by western blotting. Following a pre-clearance with IgG and protein A/G beads (Santa Cruz Biotechnology, Santa Cruz, CA, USA) at 4°C for 2 h, extracts were incubated with anti-FLAG beads (Sigma-Aldrich) overnight at 4°C. The immunoprecipitated protein complexes were eluted with FLAG peptide (Sigma-Aldrich), resolved on SDS-PAGE, and stained with Coomassie Brilliant Blue. Several differently migrating bands were excised, digested with trypsin, and analyzed by LC/MS following the reported protocol (Tang et al., 2013).

### Coimmunoprecipitation (co-IP) Assays

Cell lysates were first incubated with anti-TRIM6 (Biorbyt, Orb1893), anti-TSC1 (Cell Signaling Technology, #4906) anti-TSC2 (Cell Signaling Technology, #4308) or control IgG (Santa Cruz Biotech., Santa Cruz, CA, USA) for 1 h at 4°C and then with protein A/G-agarose for an additional 3 h at 4°C. Precipitates were washed three times in the lysis buffer and detected by western blot analysis.

### Immunofluorescence Staining

HK2 cells cultured on the coverslips were washed twice in phosphate-buffered saline (PBS), fixed in 4% paraformaldehyde for 30 min, and then blocked with 5% BSA at RT for 1 h. The cells were incubated with rabbit anti-TRIM6 (Bioss Inc., bs-9165R) and mouse anti-TSC1 (Santa Cruz, sc-514817) or mouse anti-TSC2 (Novus Biologicals, Inc.; Littleton, CO, USA, MAB40401) overnight at 4°C. Cells were washed three times with PBS and then incubated with the Alexa Fluor 555-labeled goat anti-rabbit IgG(H+L) (Beyotime Biotech.) and Alexa Fluor 488-labeled goat anti-mouse IgG(H+L) (Beyotime Biotech.) at room temperature for 1 h. After washing twice with PBS, 4′-6-diamidino-2-phenylindole (DAPI, Beyotime Biotech.) was used to stain the nuclei.

### Luciferase Reporter Assay

The full-length *TRIM6* promoter (GenBank: NM_058166) was synthesized by GeneWiz (Suzhou, China), and inserted into the firefly luciferase reporter gene vector, pGL3 (Promega, Madison, WI). HK2 cells were transfected with pGL3-TRIM6 promoter and the pRL-TK plasmid (*Renilla* luciferase, Promega) using Lipofectamine 2000 and treated as indicated. Luciferase and *Renilla* activity was determined using Dual-Luciferase® Reporter Assay kit (Promega) using a GloMax®-Multi+ Detection System. The promoter activity was calculated as the ratio between the activities of firefly to *Renilla* intensity.

### Construction of Adenovirus Expressing TRIM6 shRNAs

shRNAs targeting rat *TRIM6* and negative control (Supplementary Table 5) were designed, synthesized, annealed, and subcloned into pShuttle-H1 (GenScript). The shuttle plasmid linearized by *Pme*I was recombined with back-bone pAdEasy-1 (GenScript) in *E.coli* BJ5183 to construct pAd-shTRIM6 and pAd-shNC. pAd-shTRIM6 and pAd-shNC were then transfected into 293T cells using Lipofectamine 2000 according to the protocol provided by the manufacturer. Recombinant adenovirus Z was grown and purified with a cesium chloride gradient. After viral titers were determined by the plaque assay, the virus was aliquoted and stored at −80°C.

### Animal Model

Male Sprague–Dawley rats (8 weeks, 200–250 g) were obtained from the Shanghai Sippr-BK laboratory animal Co. Ltd. (Shanghai, China). Eighteen rats were randomly divided into three groups: Group I, sham-operated (sham)+shNC; Group II, 5/6 NX+ shNC; Group III, 5/6 NX+shTRIM6#3. The entire surgical procedure was performed under strict sterile conditions as previously described (Zhao et al., 2012). One month after surgery, recombinant adenovirus shNC (5 × 10^9^ pfu per rat, 0.5 mL) were injected into the tail vein of rats in group II, while recombinant adenovirus shNC and shTRIM6#3 (5 × 10^9^ pfu per rat, 0.5 mL) were injected into the tail vein of rats in group II and group III, respectively. Four weeks after treatment, all rats were sacrificed, and serum was collected for determination of creatinine and urea nitrogen. Kidneys were immediately excised. Some samples were fixed with 4% paraformaldehyde and others were frozen in liquid nitrogen for later use. For histological examination, renal tissues were stained with hematoxylin & eosin (HE) and Masson's trichrome. All procedures were performed as per our university's guidelines for animal care (Approval number: 2019-AR-005).

### Hydroxyproline Assay

The hydroxyproline assay was performed as per the manufacturer's product protocol (A030-2, Nanjing Jiancheng Bioengineering Institute, Nanjing, China). The urine sample (1 mL) was diluted with the solubilization solution and boiled at 95°C for 20 min. The pH of the solution was then adjusted to 6.8 followed by the incubation with 30 mg of activated carbon. The mixture was then centrifuged at 3,500 rpm for 10 min. The supernatant was saved for further tests. Distilled water, standard solution, and detection solution were added to 1 mL of the sample respectively as the blank, standard, and test groups.

Reagent I was added to the sample following 10-min incubation. Reagent II was then added and incubated for 5 min. After adding Reagent III, the sample was incubated at 60°C for 15 min and centrifuged at 3,500 rpm for 10 min. The supernatant was used for spectrophotometry at 550 nm.

### Blood Urea Nitrogen Assay

The blood urea nitrogen assay was performed as per the manufacturer's product protocol (C030-2, Nanjing Jiancheng Bioengineering Institute, Nanjing, China). Distilled water, standard solution, and detection solution as well as the buffer were added to 1 mL of the sample respectively as the blank, standard, and test groups. The sample was incubated at 37°C for 10 min. The chromogenic reagent and the sodium hypochlorite solution were added with incubation at 37°C for 10 min. The absorbance at 640 nm was detected.

### Serum Creatinine Assay

The serum creatinine assay was performed as per the manufacturer's product protocol (C011-1, Nanjing Jiancheng Bioengineering Institute, Nanjing, China). The sample was diluted with double distilled water and Reagent III and Reagent IV were sequentially added followed by incubation at 37°C for 10 min. The absorbance was detected at 510 nm.

### Statistical Analysis

Statistical analysis was carried out using Graphpad Prism software (version 6.0, San Diego, CA, USA). Student's *t*-test and analysis of variance (ANOVA) were used to compare the data. *P*-values < 0.05 were considered significant.

## Results

### TRIM6 Is Highly Expressed in Renal Fibrosis and Positively Correlated With the Severity of Renal Fibrosis

**Figure 1:**
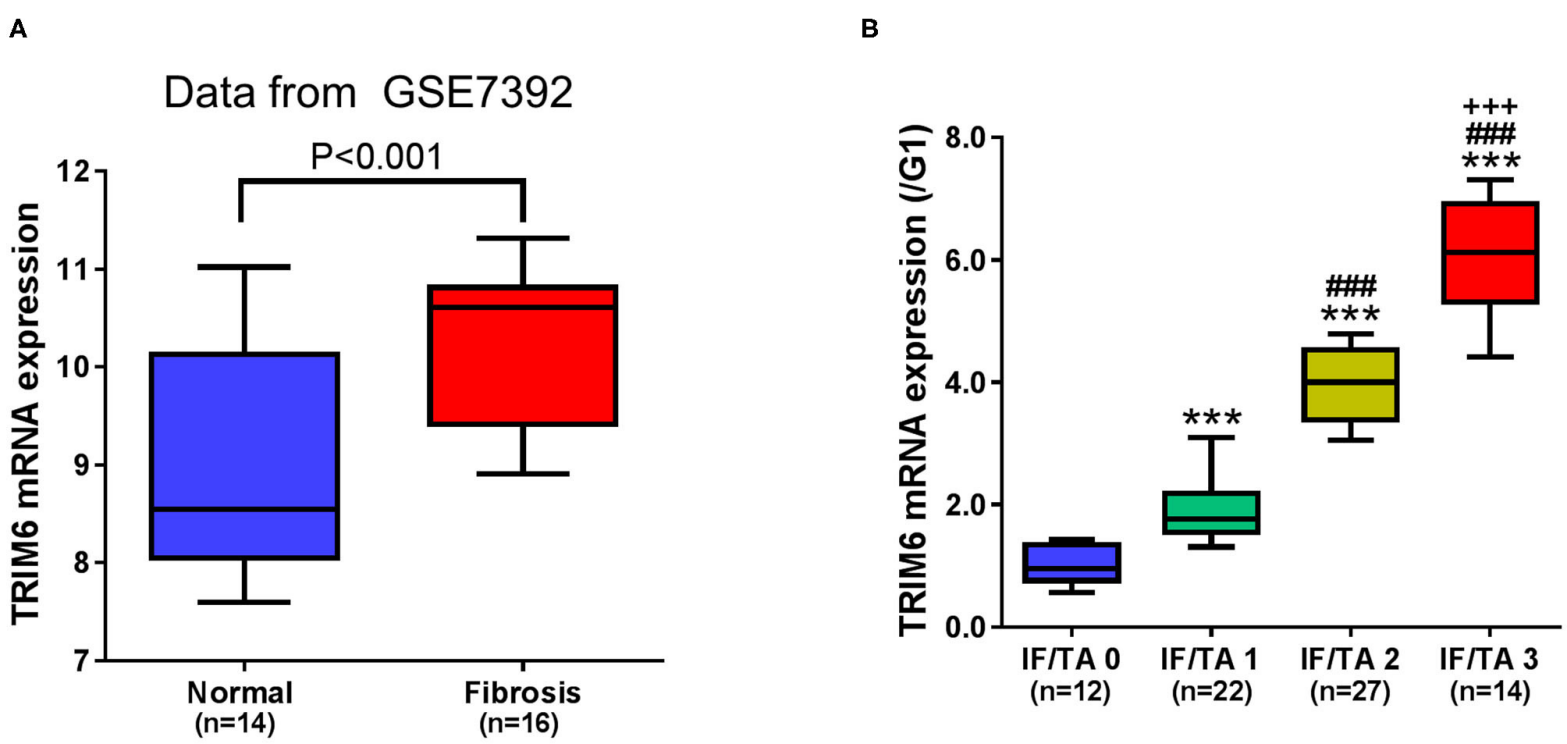
TRIM6 is highly expressed in renal fibrosis and positively correlated with the severity of renal fibrosis. **(A)** The gene expression level of TRIM6 in the GSE7392 renal fibrosis dataset from the GEO. *P* < 0.001 (*t*-test). **(B)** Based on the Banff criteria, patients with renal fibrosis were classified into IF/TA 0 to IF/TA 2 groups. The mRNA expression of TRIM6 was detected in renal fibrotic tissues by quantitative RT-PCR. ***P < 0.001 vs. IF/TA 0; ^###^*P* < 0.001 vs. IF/TA 1; ^+++^*P* < 0.001 vs. IF/TA 2 (ANOVA test).

Elevated TRIM6 has been observed in the peripheral blood samples of idiopathic pulmonary fibrosis (Li et al., 2020). We first examined the gene expression pattern of TRIM6 in renal fibrosis tissues (Figure 1). Based on the dataset of GSE7392 from the GEO database (Gene Expression Omnibus, https://www.ncbi.nlm.nih.gov/geo/), it was identified that the expression of TRIM6 in fibrosis tissues was significantly higher than that in normal tissues (Figure 1A). Using quantitative RT-PCR, we then explored the TRIM6 transcription level in the tissues obtained from 75 cases of renal fibrosis (Figure 1B). The expression of TRIM6 was proportional to the severity of renal fibrosis based on the estimated IF/TA score—TRIM6 mRNA expression in the IF/TA 3 group was ~4 times its expression in the IF/TA 0 group. These results suggested that TRIM6 may be associated with renal fibrosis.

### Inhibition of TRIM6 Ameliorates Renal Fibrosis and ER Stress

**Figure 2:**
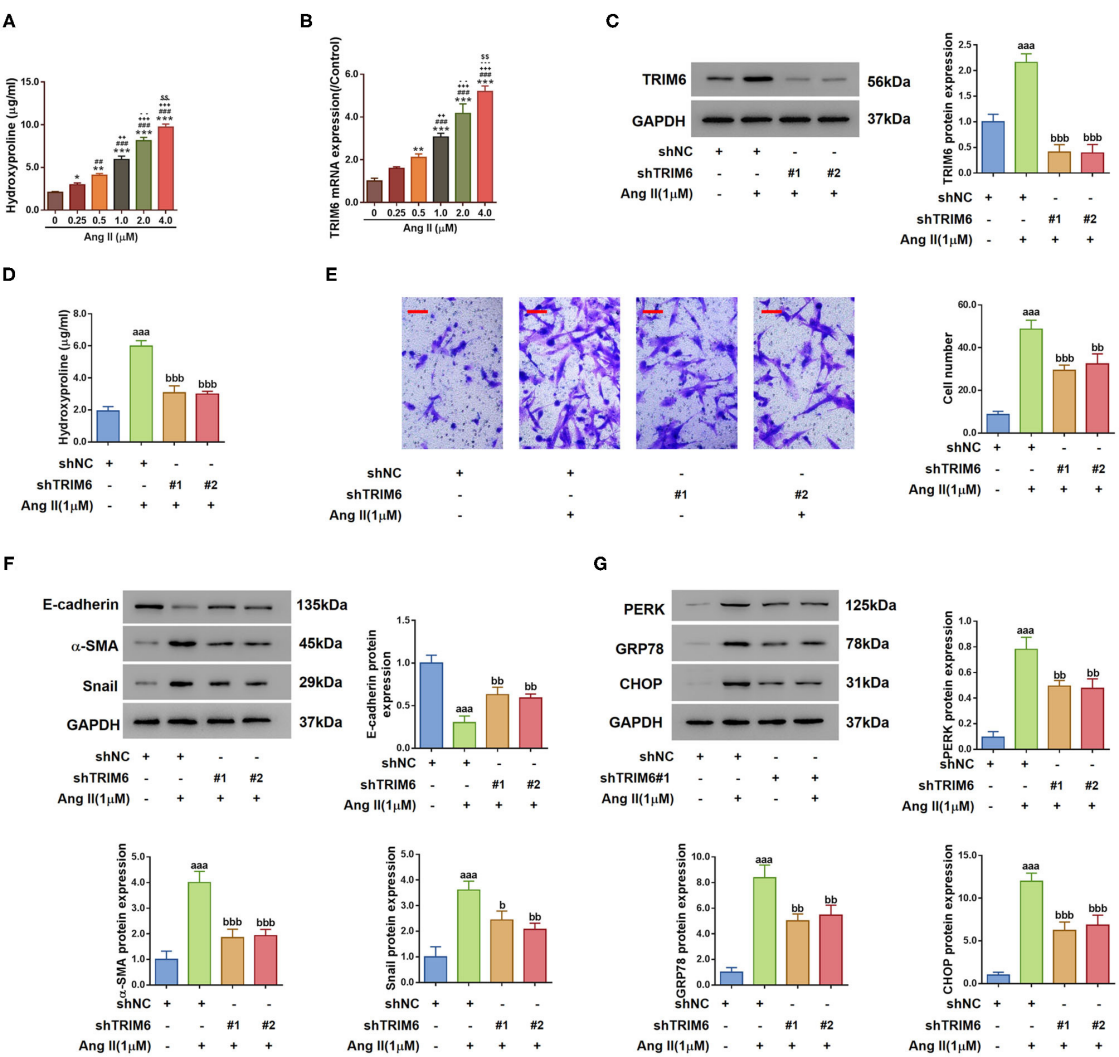
TRIM6 mediated Angiotensin II-induced EMT and ER stress in HK2 cells. **(A,B)** HK2 cells were treated with augmented concentrations of Ang II (0, 0.25, 0. 5, 1.0, 2.0, and 4.0 μM) for 24 h, the concentration of hydroxyproline in the cultured medium **(A)** and the TRIM6 transcription level **(B)** were detected by the hydroxyproline assay and quantitative RT-PCR, respectively. *P < 0.05, **P < 0.01, ***P < 0.001 vs. 0 μM; ^##^*P* < 0.01, ^###^*P* < 0.001 vs. 0.25 μM; ^++^*P* < 0.01, ^+++^*P* < 0.001 vs. 0.5 μM; ^−^*P* < 0.01, ^−−^*P* < 0.001, ^−−−^*P* < 0.001 vs. 1.0 μM; ^$$^*P* < 0.01 vs. 4.0 μM. **(C–G)** HK2 cells were transduced with TRIM6 shRNAs (shTRIM6-1#, 2#) or the control shRNA (shNC), and then treated with 1 μM of Ang II. **(C)** TRIM6 protein expression was detected by western blotting. **(D)** Hydroxyproline release in the culture medium was determined by the hydroxyproline assay. **(E)** Migration ability was assessed by Transwell assay. Scale bar: 100 μm. **(F)** Expression of EMT biomarkers was detected by western blotting. **(G)** ER stress-related proteins were detected by western blotting. ^aaa^*P* < 0.001 vs. shNC; ^b^*P* < 0.05, ^bb^*P* < 0.01, ^bbb^*P* < 0.001 vs. shNC+Ang II (ANOVA test).

To elucidate the regulatory function of TRIM6 in renal fibrosis, we treated HK2 proximal tubule epithelial cells with Angiotensin II (Ang II), which induces EMT and ER stress during the progression of chronic kidney damage, eventually contributing to renal fibrosis (Carlisle et al., 2012; Lavoz et al., 2012). Then, hydroxyproline, an amino acid specific to collagen synthesis, was used to measure fibrosis (Woessner, 1961). As shown in Figure 2A, when treated with 0–4.0 μM of Ang II, HK2 cells demonstrated augmented quantities of hydroxyproline aligning with the stimulation concentrations. The transcription of TRIM6 in HK2 cells followed the same trend with elevated Ang II concentrations (Figure 2B), substantiating the correlation between the TRIM6 expression and renal fibrosis. Ang II at the dose of 1 μM, according to previous studies (Chang et al., 2016; Li et al., 2016), was chosen for the subsequent experiments.

To investigate whether TRIM6 influence the effects of Ang II, TRIM6 expression was downregulated using shRNA toward TRIM6 (Supplementary Figure 1 and Figure 2C). As shown in Figure 2D, Ang II-increased hydroxyproline concentration was significantly dropped when TRIM6 expression was knocked down (shTRIM6#1 and #2). Ang II promoted the transition of HK2 cells from the epithelial state to the spindle-shaped mesenchymal state, while in the presence of shRNAs, the cellular morphology transition of HK2 cells was efficiently inhibited (Supplementary Figure 2). Transwell assay (Figure 2E) and detection of the cellular expression of EMT biomarkers, including E-cadherin, α-SMA, and Snail, also demonstrated an inhibited EMT process in HK2 cells with TRIM6 knockdown (Figure 2F). Similarly, despite the stimulation of HK2 cells by Ang II, the expression of effector proteins in the ER stress pathway, PERK, GRP78 and CHOP, was also lowered by the TRIM6 knockdown (Figure 2G). Together, these results reveal the important role of TRIM6 in EMT and ER stress in Ang II-treated HK2 cells.

### TRIM6 Attenuates EMT and ER Stress Through the mTORC1 Pathway

**Figure 3:**
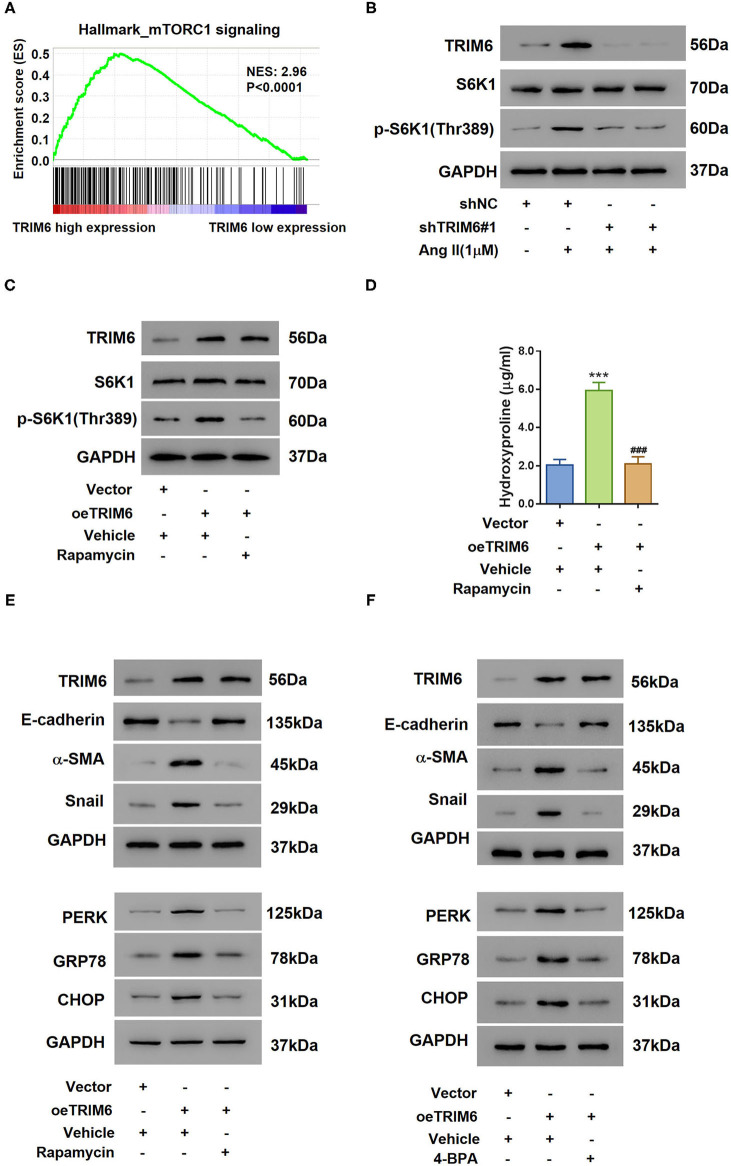
TRIM6 regulates EMT and ER stress through the mTORC1 pathway. **(A)** The GSEA results showed a positive correlation between TRIM6 expression and the mTORC1 signaling in the GSE7392 renal fibrosis dataset. **(B)** HK2 cells were transduced with TRIM6 shRNAs (shTRIM6-1#, 2#) or control shRNA (shNC) and treated with 1 μM of Ang II. The levels of S6K1 expression and phosphorylation [p-S6K1 (Thr389)] were examined by western blotting. **(C–E)** HK2 cells were transduced with lentivirus overexpressing (oeTRIM6) or control Vector, and then exposed to 100 nM of Rapamycin or Vehicle. S6K1 phosphorylation **(C)**, the concentration of hydroxyproline **(D)**, and the expression of biomarkers of EMT and ER stress **(E)** were detected. ***P < 0.001 vs. Vector+Veh; ^###^*P* < 0.001 vs. oeTRIM6+Veh (ANOVA test). **(F)** HK2 cells were transduced with lentivirus overexpressing (oeTRIM6) or control Vector, and then exposed to 2 mM 4-BPA or Vehicle. The expression of biomarkers of EMT and ER stress was examined by western blotting.

To further examine the pathway TRIM6 participated in, Gene Set Enrichment Analysis (GSEA) was performed with the GSE7392 renal fibrosis dataset (Figure 3A). Interestingly, we identified a positive correlation between the TRIM6 expression and the mTORC1 signaling. It has been reported that Ang II can induce the phosphorylation of S6K1, an indication of mTORC1 activation (Muta et al., 2016). When treated with shRNAs targeting TRIM6, even in the presence of Ang II, HK2 cells exhibited reduced phosphorylation of S6K1 (Thr389) without changing the protein level of S6K1 (Figure 3B), suggesting that the knockdown of TRIM6 counteracted the activation of the mTORC1 signaling. By contrast, the overexpression of TRIM6 in HK2 cells aided in S6K1 phosphorylation (Thr389), demonstrating a robustly activated mTORC1 pathway, while rapamycin was able to abolish this activation (Figure 3C). Overexpression of TRIM6 showed the same effects as Ang II on the phosphorylation of S6K1, release of hydroxyproline, as well as the expression of EMT and ER stress-related proteins, which were tremendously blocked in the presence of rapamycin (Figures 3C–E). Similarly, using 4-phenylbutyric acid (4-PBA), an ER stress inhibitor, we also observed inhibited EMT and ER stress in TRIM6-overexpressing HK2 cells (Figure 3F). These findings manifest that TRIM6 facilitates downstream processes of ER stress and EMT *via* the mTORC1 signaling.

### TRIM6 Participates in the mTORC1 Signaling Through Activating the Ubiquitination of TSC1 and TSC2

**Figure 4:**
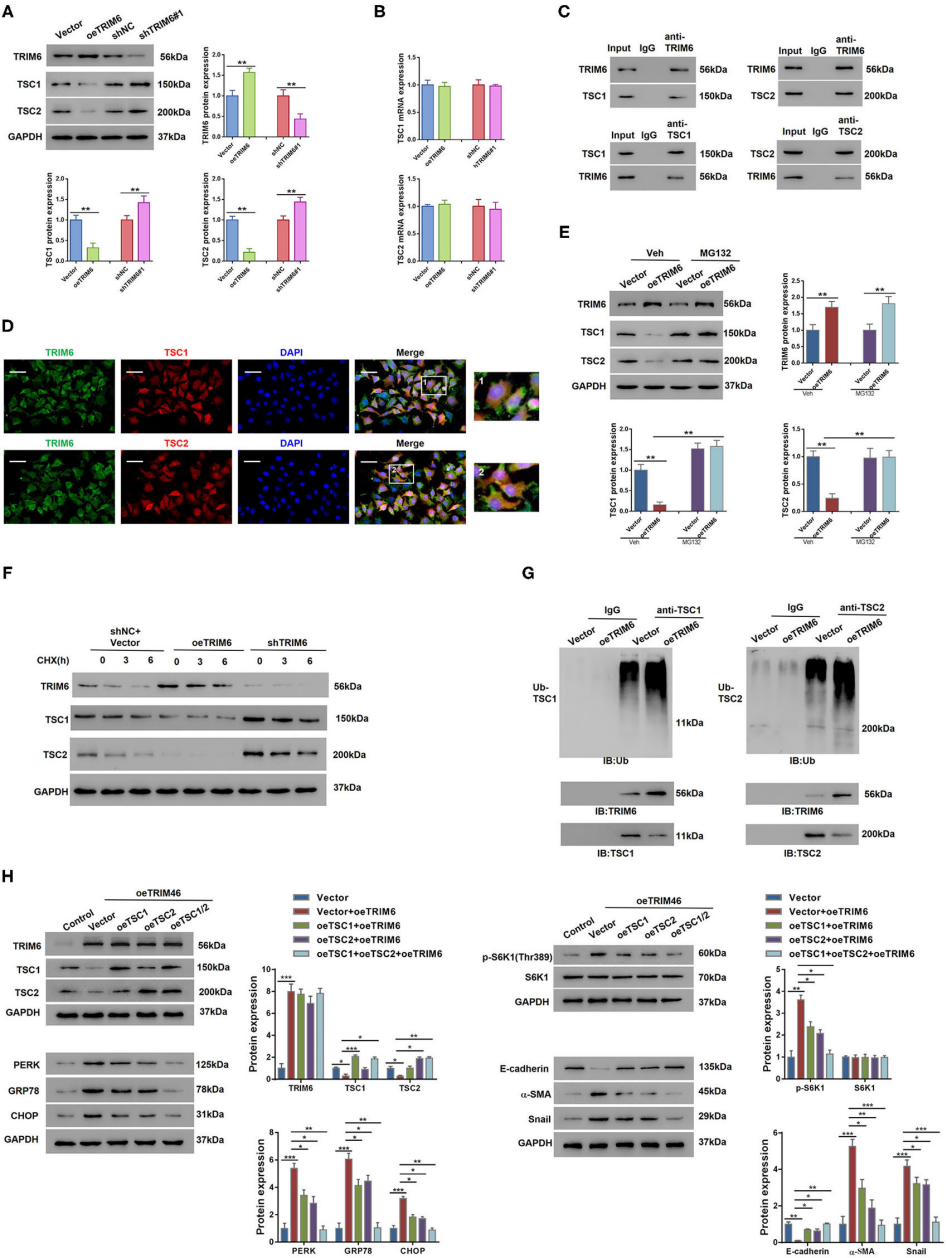
TRIM6 interacts with TSC1 and TSC2 to participate in the mTORC1 signaling. **(A,B)** The protein levels **(A)** and the mRNA levels **(B)** of TSC1 and TSC2 when TRIM6 was knocked down and overexpressed in HK2 cells. **(C)** Co-immunoprecipitation analysis of the interactions between TRIM6 and TSC1/2. **(D)** The cellular colocalization of TRIM6 and TSC1/2. TRIM6 in green, TSC1/2 in red, and DAPI in blue. Scale bar: 50 μm. Magnified view fields are shown at the right. **(E)** The protein levels of TSC1 and TSC2 in TRIM6-overexpressing HK2 cells with or without the treatment of 10 μM of MG132. **(F)** HK2 cells were transduced with lentivirus expressing TRIM6 (oeTRIM6), shTRIM6, or shNC+Vector for 24 h, and exposed to 20 mM cycloheximide (CHX, Sigma-Aldrich) at 0, 3, and 6 h after exposure. The expression of TRIM6, TSC1, and TSC2 was detected by western blotting analysis. **(G)** Cell lysates from HK2 cells infected with lentivirus overexpressing TRIM6, or Vector were IP with anti-TSC1, anti-TSC2, or control IgG, and then immunoblotted for ubiquitin (Ub). **(H)** HK2 cells overexpressed with TRIM6, TSC1, and/or TSC2. The levels of TRIM6, TSC1, TSC2, S6K1, p-S6K1 (Thr389), EMT biomarkers, and ER biomarkers were examined by western blotting. *P < 0.05, **P < 0.01, ***P < 0.001 (ANOVA test).

To identify the TRIM6-interacting proteins in the mTORC1 signaling, we performed mass spectrometry-based immuno-precipitation proteomics, in which TSC1 and TSC2 ranked high among the candidate proteins that interact with TRIM6 (Supplementary Table 6 and Supplementary Figure 3). An in-depth exploration of the relationship between TRIM6 and TSC1 and TSC2 was further conducted in HK2 cells *in vitro* (Figure 4A). According to our results, TRIM6 overexpression downregulated the protein levels of TSC1 and TSC2 while the TRIM6 shRNA recovered their cellular levels. This regulation was irrelevant to changes in the transcription level of the two proteins (Figure 4B). Moreover, the results of the Co-IP and immunofluorescence staining both indicated that TRIM6 was tightly associated with TSC1 and TSC2 (Figures 4C,D). MG132, a proteasome inhibitor, was able to restore the decreased levels of TSC1 and TSC2 in cells overexpressing TRIM6, suggesting that TRIM6 mediated the proteasome-dependent degradation of TSC1 and TSC2 (Figure 4E). To determine whether TRIM6 overexpression altered the stability of TSC1 and TSC2, protein synthesis was blocked by cycloheximide (CHX, 20 mM). The results showed that the half-life of TSC1 and TSC2 protein was reduced by TRIM6 overexpression and elevated by TRIM6 knocking down (Figure 4F). To better clarify the role of TRIM6 in regulating TSC1 and TSC2, we examined the ubiquitination of the two proteins in TRIM6-overexpressing cells (Figure 4G). Notably, the ubiquitination of both proteins increased when TRIM6 was overexpressed. On the other hand, the overexpression of TSC1 and TSC2 in HK2 cells was found to synergically inhibit the TRIM6-induced processes of ER stress and EMT (Figure 4H and Supplementary Figure 4), which reinforces our speculation that TRIM6 plays a role in the mTORC1 pathway.

### The Angiotensin II-Activated NF-κB Signaling Facilitates the Transcription of *TRIM6*

**Figure 5:**
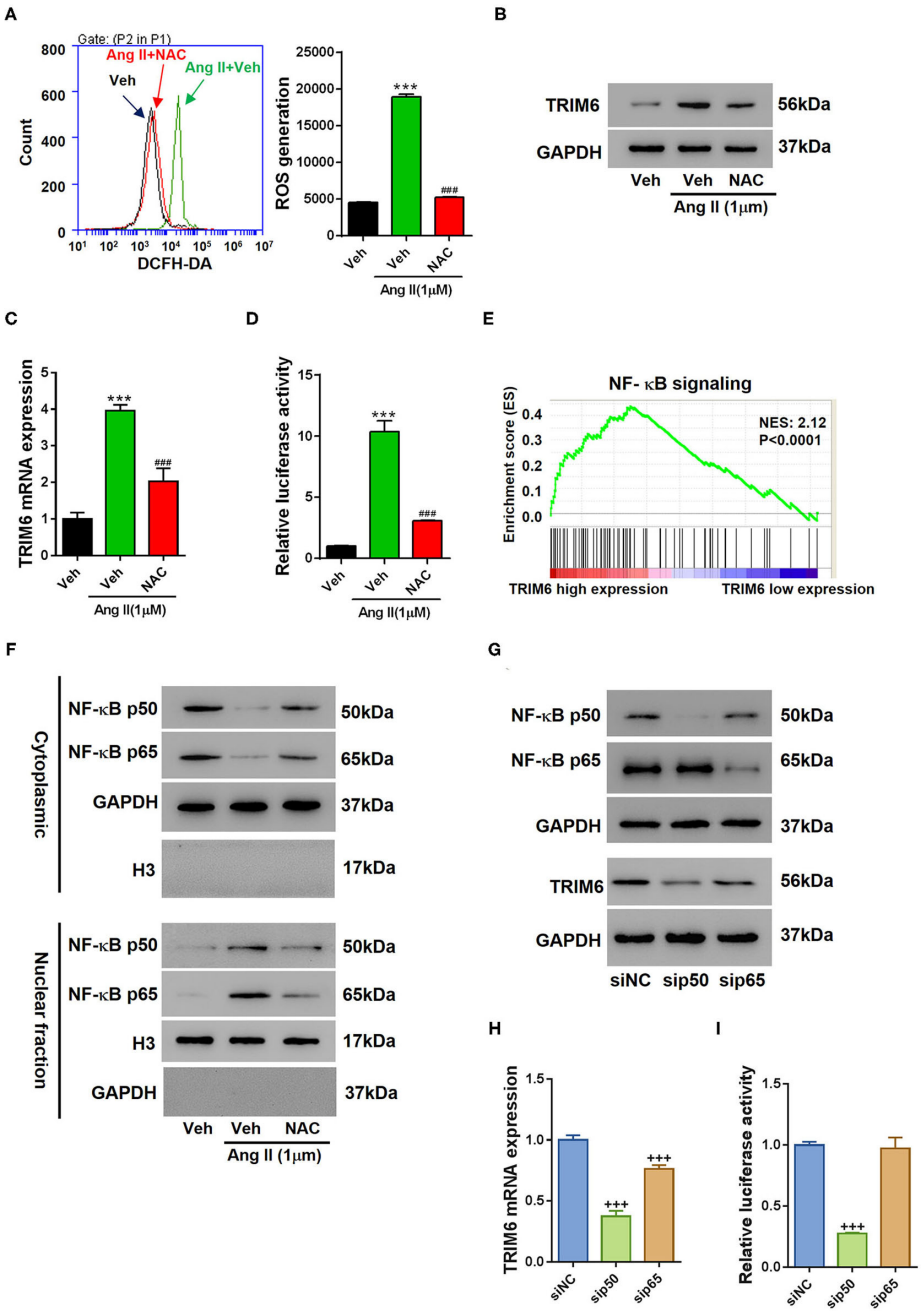
Angiotensin II-induced ROS production activated the NF-κB pathway to facilitate the transcription of *TRIM6*. **(A–D)** 100 μM of NAC or the control reagent (Veh) was applied to Ang II-treated HK2 cells. **(A)** Flow cytometry analysis of ROS accumulation in Ang II-treated HK2 cells. **(B–D)** The effects of NAC on *TRIM6* expression in Ang II-treated HK2 cells. NAC downregulated the protein level **(B)**, transcription **(C)**, and promoter activity **(D)** of *TRIM6*. **(E)** The correlation between *TRIM6* expression and the NF-κB signaling as revealed by the GSEA. **(F)** The effects of NAC on Ang II-induced nuclear translocation of p50 and p65. Knockdown of either p50 or p65 suppressed the protein level **(G)**, mRNA level **(H)**, and promoter activity **(I)** of *TRIM6*. ***P < 0.001 vs. Veh; ^*###*^*P* < 0.001 vs. Veh+Ang II. ^+++^*P* < 0.001 vs. siNC (ANOVA test).

Considering the expression of *TRIM6* is critical to renal fibrosis, we then investigated how *TRIM6* expression is regulated in HK2 cells. Ang II can induce the formation of reactive oxygen species (ROS) that account for the development of kidney diseases (Benigni et al., 2009). We added NAC (N-acetyl-L-cysteine), a ROS scavenger, to Ang II-treated HK2 cells to examine the effect of ROS accumulation on *TRIM6* expression (Figures 5A,B). NAC significantly decreased the ROS accumulation (Figure 5A) and accordingly suppressed the expression of *TRIM6* (Figure 5B), suggesting an important role of ROS in the regulation of *TRIM6* expression. Further exploration showed the transcription of *TRIM6* was suppressed by NAC treatment (Figures 5C,D), with both absolute mRNA level and promoter activity of *TRIM6* being significantly inhibited. These results indicate that ROS can regulate *TRIM6* expression at the transcription level. Considering that ROS can activate the NF- κB signaling (Lingappan, 2018), we queried the GSEA and found that the expression of *TRIM6* in renal fibrosis was tightly correlated with the NF-κB signaling (Figure 5E). Additionally, a putative binding site for NF- κB was predicted in TRIM6 promoter by using the ALGGEN PROMO software program (http://alggen.lsi.upc.es). Upon Ang II treatment, the nuclear translocation of p50 and p65 was actively driven to control the transcription of *TRIM6* (Figure 5F). Accordingly, the knockdown of either p50 or p65, especially p50, had led to a decline in protein level (Figure 5G and Supplementary Figure 5), transcription (Figure 5H), and promoter activity (Figure 5I) of *TRIM6*. Therefore, the expression of *TRIM6* was regulated by the Ang II-activated NF-κB pathway.

### Knockdown of *TRIM6* Attenuates Renal Fibrosis in 5/6 NX Rats

**Figure 6:**
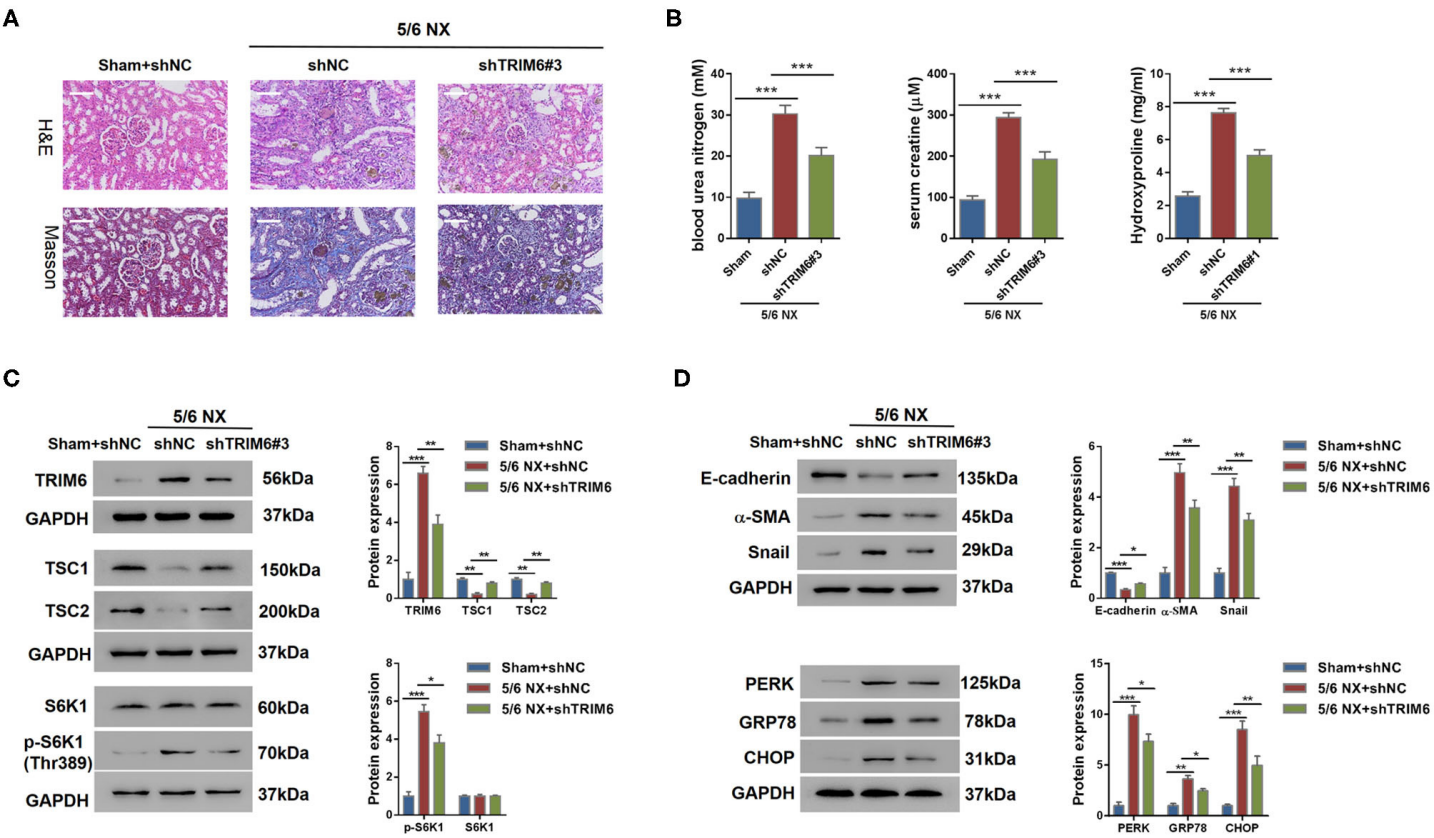
*TRIM6* knockdown attenuated renal fibrosis in 5/6 NX rats. Eighteen rats were randomly divided into three groups: Group I, sham-operated (sham)+shNC; Group II, 5/6 NX+ shNC; Group III, 5/6 NX+shTRIM6#3. One month after surgery, recombinant adenovirus shNC were injected into the tail vein of rats in group 1. Recombinant adenovirus shNC and shTRIM6#3 were injected into the tail vein of rats in group II and group III, respectively. Four weeks after treatment, all rats were sacrificed. **(A)** H&E and Masson staining results of the renal tissues with or without shTRIM6 treatment. Scale bar: 100 μm. **(B)** Concentrations of blood urea nitrogen, serum creatine, and hydroxyproline in the intervened 5/6 NX rats. **(C)** The effects of TRIM6 knockdown on TSC1 and TSC2 protein levels and the phosphorylation of S6K1 in the kidney of 5/6 NX rats. The levels of TSC1/2 and S6K1 phosphorylation [p-S6K1 (Thr389)] were examined by western blotting. **(D)** The protein levels of EMT and ER stress biomarkers in the kidneys of 5/6 NX rats under the treatment of shTRIM6 by western blot. *P < 0.05, **P < 0.01, ***P < 0.001 (ANOVA test).

Evidenced by all the aforementioned *in vitro* assay results, we believed that the expression of *TRIM6* was closely correlated with renal fibrosis. Therefore, we used the 5/6 NX rat as an *in vivo* model to study the effect of *TRIM6* knockdown on renal fibrosis. The entire surgical procedure of 5/6 NX and sham operation was performed as previously described (Zhao et al., 2012). The most potent shRNA targeting *TRIM6* (shTRIM6#3) was selected in NRK-52E cells (Supplementary Figure 6). One month after 5/6 NX, shTRIM6#3 virus, or control shRNA (shNC) virus was injected into the tail vein of rats. The rats with sham operation were injected with shNC virus. Four weeks after treatment, kidney tissues were sampled from different treatment groups for hematoxylin and eosin (HE) and Masson stainings according to the established protocols (Zhao et al., 2012) (Figure 6A). The shTRIM6#3-treated group demonstrated alleviated renal fibrosis. Consistent with the staining results, the concentrations of kidney disease biomarkers, including blood urea nitrogen, serum creatine, and hydroxyproline, were strongly reduced in the shTRIM6#3-treated group (Figure 6B). Collectively, these results indicate that knockdown of *TRIM6* can alleviate renal fibrosis in 5/6 NX rats. Consistent with the findings of the *in vitro* assays, the mTORC1 pathway was robustly attenuated by *TRIM6* knockdown (Figure 6C), thereby resulting in suppressed downstream processes of ER stress and EMT (Figure 6D). These data suggest that knockdown of *TRIM6* using shRNA that targeted the *TRIM6* gene effectively attenuated renal fibrosis *in vivo*.

### The Levels of TRIM6, TSC1, TSC1, and Nuclear NF-κB p50 Are Clinically Related to the Severity of Renal Fibrosis

**Figure 7:**
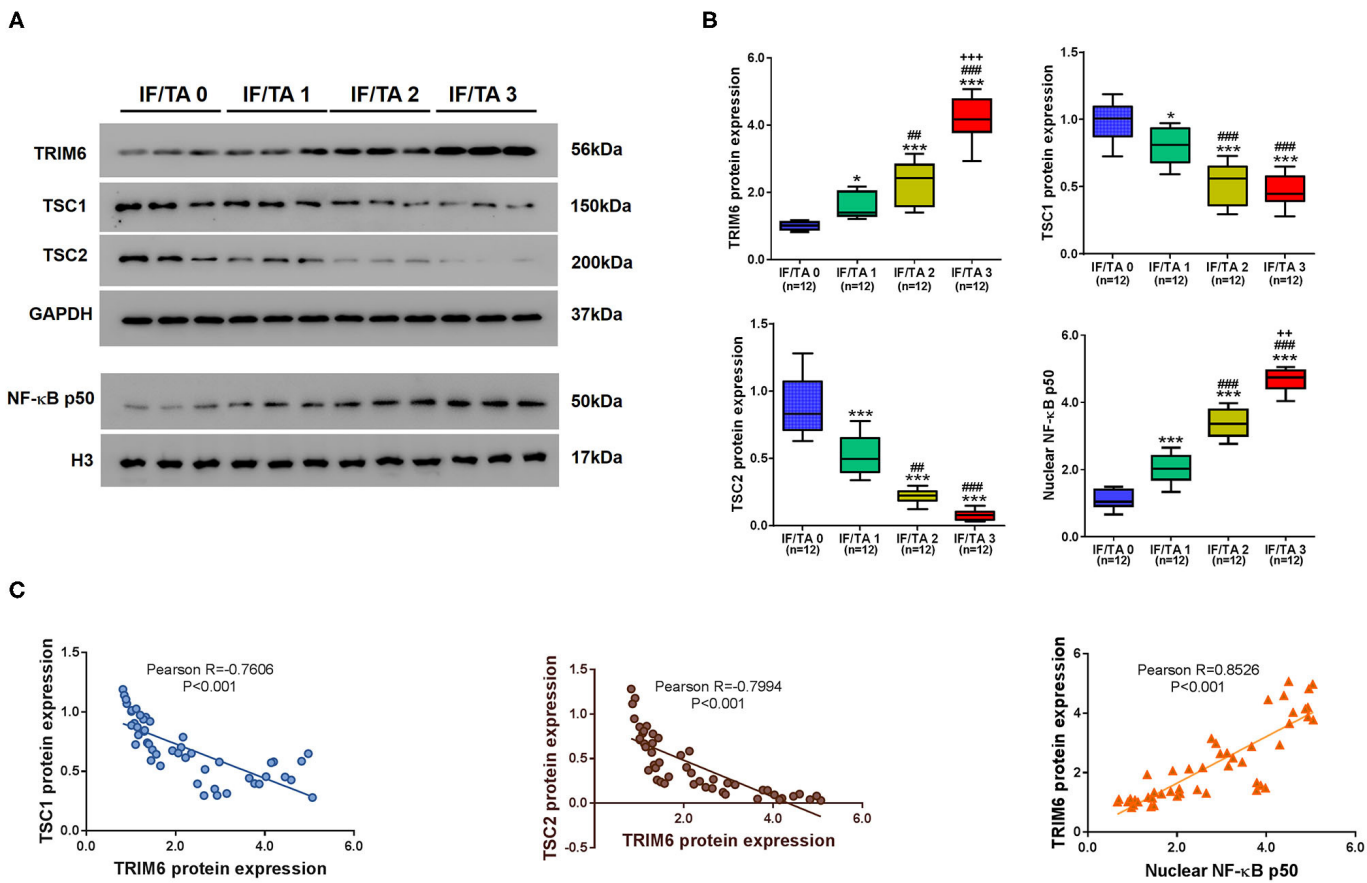
The levels of TRIM6, TSC1, TSC2, and nuclear NF-κB p50 are clinically related to the severity of renal fibrosis. **(A,B)** The levels of TRIM6, TSC1/2, and the nuclear fraction of NF-κB in the renal tissues of patients suffering renal fibrosis. **(A)** The protein levels of TRIM6, TSC1, TSC2, and nuclear NF-κB p50 in patients' renal tissues examined by western blot. **(B)** Correlations between the tissue protein quantitation of TRIM6, TSC1, TSC2, and nuclear NF-κB p50 and IF/TA scores. *P < 0.05, ***P < 0.001 vs. IF/TA 0; ^*##*^*P* < 0.01, ^###^*P* < 0.001 vs. IF/TA 1; ^++^*P* < 0.01, ^+++^*P* < 0.001 vs. IF/TA 2 (ANOVA test). **(C)** Correlations among the protein levels of TRIM6, TSC1, TSC2, and nuclear p50 in renal fibrosis tissues.

Given the roles of TRIM6, TSC1, TSC2, and nuclear NF-κB p50 in renal fibrosis, we explored the relationships between the levels of TRIM6, TSC1, TSC1, and nuclear NF-κB p50 and severity of renal fibrosis based on the estimated IF/TA scorein patients suffering from renal fibrosis of different severities (Figure 7). With the severity of renal fibrosis score, the protein levels of TRIM6 and the nuclear translocation of p50 increased significantly, while the protein levels of TSC1 and TSC2 decreased significantly in the kidney tissues of the patients (Figures 7A,B). Pearson correlation analysis was then performed to elucidate the correlations among the protein levels of TRIM6, TSC1, TSC2, and nuclear NF-κB p50 in renal tissues of the patients (Figure 7C). According to our results, the protein level of TRIM6 is negatively correlated with the protein levels of TSC1 and TSC2 but positively correlated with the nuclear translocation of p50. In summary, these clinical data support our findings in both *in vitro* and *in vivo* models.

## Discussion

**Figure 8:**
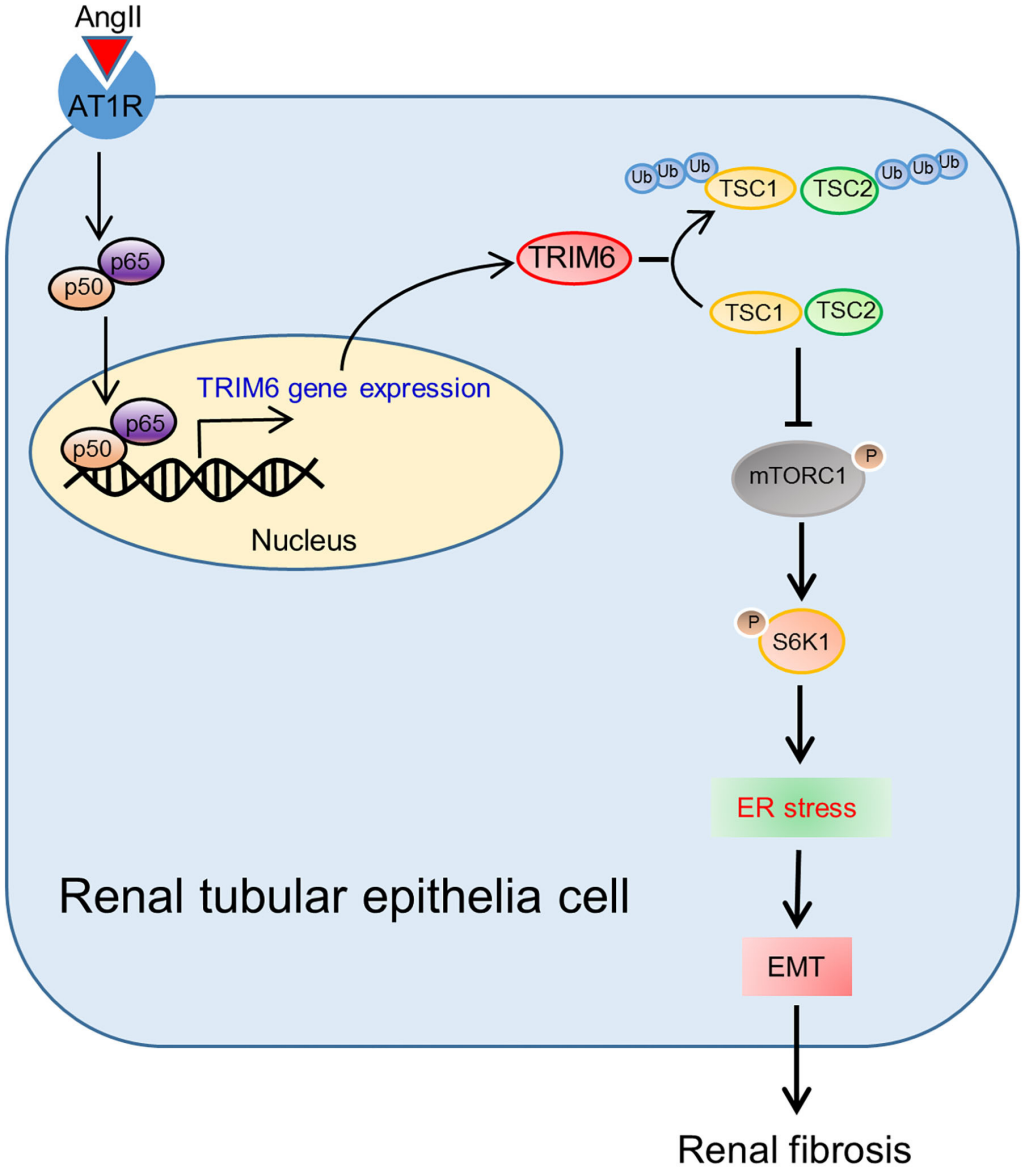
Schematic of the TRIM6-involved regulation of renal fibrosis.

Renal fibrosis, a direct consequence of reduced kidney capacity due to damages or injuries, occurs in all types of kidney diseases, which leads to a progressive loss of kidney functions and even kidney failure (Humphreys, 2018). There are only limited studies on the role of TRIM family members on kidney diseases (Duann et al., 2015; Xiao et al., 2018; Chen et al., 2020). In our research, we found that TRIM6 expression was positively correlated with the severity of renal fibrosis. Moreover, we have for the first time clarified the relationship between TRIM6 and renal fibrosis in different models: The Ang II-induced formation of ROS favored the nuclear translocation of NF-κB p50 and p65 to activate the transcription of TRIM6, which further activated the mTORC1 signaling by promoting the ubiquitination of two TSC proteins (Figure 8).

Numerous pathways, such as TGF-β1/Smads, PDGF, Wnt/β-catenin, and mTOR signalings, have been implicated in renal fibrosis (Lieberthal and Levine, 2009; Liu, 2011; Fantus et al., 2016; Nogueira et al., 2017). Increasing evidence has supported that members of the TRIM protein are involved in renal fibrosis-related pathways. For example, TRIM33 (TIF1γ) functions as a ubiquitin ligase for Smad4 and negatively regulates TGF-β signaling (Dupont et al., 2005). TRIM27 and TRIM28 form a complex, which regulates the expression of PDGF receptor-β (PDGFRβ) in vascular smooth muscle cells (Wang et al., 2020). TRIM65 activates the Wnt/β-catenin signaling pathway *via* ubiquitination of Axin1 (Yang et al., 2017). TRIM14 induces the activation of the AKT/mTOR pathway in osteosarcoma cells (Xu G. et al., 2017). In this study, we confirm the role of TRIM6 as a key upstream regulator of the mTORC1 pathway. Furthermore, the mTORC1 signaling is able to stimulate the synthesis of proteins, lipids and nucleotides, thus inhibiting autophagy (Yuan et al., 2013). Autophagy dysfunction has been implicated in the pathogenesis of renal fibrosis (Zhao et al., 2019; Tang et al., 2020). TRIM proteins including TRIM6 (Mandell et al., 2014; Di Rienzo et al., 2020) have been link to the induction of autophagy. The current study confirmed that TRIM6 overexpression resulted in higher TSC1 and TSC2 ubiquitination levels, which facilitated the proteasome-dependent degradation of the two proteins and led to the activation of the mTORC1 pathway. Previous study has shown that the TSC1/2 complex translocate to lysosomes to inactivate mTORC1 in response to amino-acid starvation and growth factor removal (Demetriades et al., 2016). Whether TRIM6 alters the translocaiton capacity of TSC1/2 will be investigated in the future.

More importantly, the suppressed expression of TRIM6 by shRNAs largely attenuated renal fibrosis *in vitro* and *in vivo*, suggesting that TRIM6 can be used as a potential therapeutic target for renal fibrosis. Due to the lack of successful cases of treating renal fibrosis using shRNAs, our work definitely fills this knowledge gap. Our results also represent the first attempt to treat renal fibrosis from the perspective of the TRIMosome. We prove that the disruption of TRIMosome formation tremendously affects the progression of renal fibrosis. As a result, other components of the TRIMosome can be therapeutic targets for renal fibrosis.

The revelation of the important regulatory role of ROS and the NF-κB pathway in TRIM6 expression in our study contributes to a better understanding of the entire picture of TRIM6-related signaling pathways in renal fibrosis. ROS in cells plays various inhibitory or stimulatory roles in the NF-κB signaling, which mediates oxidation of upstream kinases to influence the NF-κB pathway (Lingappan, 2018). For example, ROS markedly decreased the ability of TNF to induce IKK activity, resulting in NF-κB activation (Korn et al., 2001). The production of ROS induced by Ang II promoted the nuclear translocation of NF-κB p50 and p65 to facilitate the transcription of the *TRIM6* gene. TRIM6 overexpression further led to the activation of downstream processes of EMT and ER stress that contribute to renal fibrosis. As a result, these findings reasonably explain how Ang II induces renal fibrosis in HK2 cells, indicating that TRIM6 is an important but previously unrevealed piece of the puzzle.

Considering that the number of current studies on TRIM6 still remains limited, more sophisticated investigations should be devised and conducted on TRIM6 to explore its precise molecular functions. Based on our mass spectrometry results, it is promising to identify more TRIM6-interacting proteins, which will help better understand the functions of TRIM6. Likewise, further characterization of TRIM6 as an mTORC1 pathway effector or regulator protein is likely to unveil the mystery behind. The establishment of a TRIM6-involved regulatory network concerning the ubiquitination of TSC1 and TSC2 can also be a breakthrough to elucidate the entire picture of the signaling pathway.

Paces for discovering the mechanism of renal fibrosis have never stopped. Motivated by the goal of developing novel therapies for kidney diseases, researchers have made great efforts to investigate potential solutions (Klinkhammer et al., 2017; Liu and Zhuang, 2019; Wakui et al., 2020). Our study has uncovered a TRIM6-involved regulatory mechanism of renal fibrosis through the mTOCR1 signaling pathway for the first time. Our work blazes a new way for a potential therapeutic solution for treating renal fibrosis using TRIM6 inhibitors. With the development of the technologies of drug delivery, different forms of inhibitors targeting TRIM6, i.e., shRNAs and neutralizing antibodies, can be internalized into kidney tissues and cells to suppress TRIM6 expression and treat renal fibrosis in patients with kidney diseases.

## Data Availability Statement

Publicly available datasets were analyzed in this study. This data can be found at: https://www.ncbi.nlm.nih.gov/geo/query/acc.cgi?acc=GSE7392.

## Ethics Statement

The studies involving human participants were reviewed and approved by the ethics committee of Seventh People's Hospital of Shanghai University of Traditional Chinese Medicine (Shanghai, China). The patients/participants provided their written informed consent to participate in this study. The animal study was reviewed and approved by Ethics committtee of Seventh People's Hospital of Shanghai University of Traditional Chinese Medicine.

## Author Contributions

WeiL, JC, and JL designed the experiments. WeiL, YY, CZ, BZ, LL, and WenL performed the experiments. WeiL, JH, and QX performed the statistical analysis. WeiL and YY wrote the manuscript. JC and JL supervised the study. All authors have read and approved the final version of the manuscript.

## Conflict of Interest

The authors declare that the research was conducted in the absence of any commercial or financial relationships that could be construed as a potential conflict of interest.
