# Supplementary material for: The Expression of TRIM6 Activates the mTORC1 Pathway by Regulating the Ubiquitination of TSC1-TSC2 to Promote Renal Fibrosis
Source: Front Cell Dev Biol. 2021 Feb 9;8:616747. doi: 10.3389/fcell.2020.616747 (PMC7901959; doi:10.3389/fcell.2020.616747)
Supplement: Supplementary file 1 [file Table_1.DOCX]

**Table S1.** Primer sequences for real-time PCR.

| **Gene** | **Forward primer** | **Reverse primer** |
| --- | --- | --- |
| TRIM6 | 5' CTTTCCCACTACTCTTTGTC 3' | 5' TAAGCCTCAGGGTACTTATC 3' |
| TSC1 | 5' CGAAGGTGGAAGAGATTAG 3' | 5' GTGGTGGTTCAGTTATCAG 3' |
| TSC2 | 5' GAAAGTGCCAGGCATCAACC 3' | 5' TACAGGACGGCGATCTTGTG 3' |
| GAPDH | 5' AATCCCATCACCATCTTC 3' | 5' AGGCTGTTGTCATACTTC 3' |

**Table S2.** Antibody list.

| **Primary antibody** | **Company** | **Catalog No.** |
| --- | --- | --- |
| TRIM6 | Proteintech | 11953-1-AP |
| PERK | Abcam | Ab65142 |
| GRP78 | Abcam | Ab108613 |
| CHOP(DDIT3) | Abcam | Ab11419 |
| E-cadherin | Cell Signaling Technology | #14472 |
| α-SMA | Abcam | Ab5694 |
| Snail | Abcam | Ab216347 |
| S6K1 | Abcam | Ab186753 |
| p-S6K1 (Thr389) | Cell Signaling Technology | #9234 |
| TSC1 | Abcam | Ab247297 |
| TSC2 | Cell Signaling Technology | #3612 |
| Ubiquitin | Abcam | Ab7780 |
| GAPDH | Cell Signaling Technology | #5174 |

**Table S3.** Target sequences of human TRIM6 shRNAs.

| **shRNA** | **Target sequence** |
| --- | --- |
| shTRIM6#1 | 5’CCGGAGACAAGTGAGGTTT 3’ |
| shTRIM6#2 | 5’GCTAAAGTATCTGGACCTT 3’ |
| shTRIM6#3 | 5’CCATGAATATAGGGCCTAT 3’ |

**Table S4.** siRNA sequences.

| **siRNA** | **Sequence** |
| --- | --- |
| sip65#1 | Sense: 5’- GGAGCACAGAUACCACCAATT -3’  Antisense: 5’-UUGGUGGUAUCUGUGCUCCTT-3’ |
| sip65#2 | Sense: 5’- UCUUCCUACUGUGUGACAATT -3’  Antisense: 5’-UUGUCACACAGUAGGAAGATT-3’ |
| sip65#3 | Sense: 5’- GGACAUAUGAGACCUUCAATT -3’  Antisense: 5’-UUGAAGGUCUCAUAUGUCCTT-3’ |
| sip50#1 | Sense: 5’- GCAAUCAUCCACCUUCAUUTT -3’  Antisense: 5’-AAUGAAGGUGGAUGAUUGCTT-3’ |
| sip50#2 | Sense: 5’- GCCAGAGUUUACAUCUGAUTT -3’  Antisense: 5’-AUCAGAUGUAAACUCUGGCTT-3’ |
| sip50#3 | Sense: 5’- GCUGCAGCUGUAUAAGUUATT -3’  Antisense: 5’-UAACUUAUACAGCUGCAGCTT-3’ |
| siNC | Sense: 5’- UUCUCCGAACGUGUCACGUTT -3’  Antisense: 5’-ACGUGACACGUUCGGAGAATT-3’ |

**Table S5.** Target sequences of rat TRIM6 shRNAs.

| **shRNA** | **Target sequence** |
| --- | --- |
| shTRIM6#1 | 5’ GACAAGAGAGTGACTACAA 3’ |
| shTRIM6#2 | 5’ GCTCAGAGAAGTCGTGTTA 3’ |
| shTRIM6#3 | 5’ GGATCCAGACAGAGTTTAA 3’ |

**Table S6.** List of proteins immunoprecipitated with anti-Flag and identified by mass spectrometry.

| Accession | Description | Score | Coverage | # Proteins | # Unique Peptides | # Peptides | # PSMs | # AAs | MW [kDa] | calc. pI |
| --- | --- | --- | --- | --- | --- | --- | --- | --- | --- | --- |
| Q14624 | Inter-Alpha-Trypsin Inhibitor Heavy Chain 4 OS=Homo sapiens GN=ITIH4 PE=1 SV=2 - [ITIH4_HUMAN] | 839.57 | 65.49 | 2 | 35 | 35 | 60 | 339 | 103.4 | 6.97 |
| Q8N3V7 | Synaptopodin OS=Homo sapiens GN=SYNPO PE=1 SV=3 - [SYNPO_HUMAN] | 1331.58 | 66.27 | 1 | 29 | 30 | 91 | 335 | 99.5 | 8.98 |
| Q92574 | TSC Complex Subunit 1 OS=Homo sapiens GN=TSC1 PE=1 SV=2 - [TSC1_HUMAN] | 1016.69 | 60.12 | 1 | 29 | 29 | 47 | 346 | 129.8 | 6.44 |
| Q9UI36 | Dachshund Family Transcription Factor 1 OS=Homo sapiens GN=DACH1 PE=1 SV=2 - [DACH1_HUMAN] | 1477.98 | 50.82 | 1 | 27 | 27 | 56 | 364 | 78.6 | 8.72 |
| Q96QB1 | DLC1 Rho GTPase Activating Protein OS=Homo sapiens GN=DLC1 PE=1 SV=2 - [DLC1_HUMAN] | 866.77 | 42.25 | 6 | 19 | 27 | 35 | 639 | 170.6 | 6.35 |
| Q14839 | Chromodomain Helicase DNA Binding Protein 4 OS=Homo sapiens GN=CHD4 PE=1 SV=5 - [CHD4_HUMAN] | 709.53 | 39.54 | 6 | 3 | 26 | 34 | 564 | 218.0 | 5.63 |
| Q15418 | Ribosomal Protein S6 Kinase A1 OS=Homo sapiens GN=RPS6KA1 PE=1 SV=3 - [RPS6KA1_HUMAN] | 569.33 | 35.93 | 4 | 16 | 26 | 33 | 590 | 82.7 | 7.94 |
| Q9UPQ9 | Trinucleotide Repeat Containing Adaptor 6B OS=Homo sapiens GN=TNRC6B PE=1 SV=2 - [TNRC6B_HUMAN] | 1051.57 | 66.33 | 2 | 25 | 25 | 52 | 294 | 194.0 | 6.73 |
| P49815 | TSC Complex Subunit 2 OS=Homo sapiens GN=TSC2 PE=1 SV=3 - [TSC2_HUMAN] | 790.07 | 51.69 | 1 | 24 | 25 | 38 | 623 | 200.6 | 7.33 |
| Q6PKG0 | La Ribonucleoprotein 1, Translational Regulator OS=Homo sapiens GN=LARP1 PE=1 SV=2 - [LARP1_HUMAN] | 744.45 | 42.13 | 11 | 13 | 23 | 36 | 432 | 123.5 | 9.25 |
| P09493 | Tropomyosin alpha-1 chain OS=Homo sapiens GN=TPM1 PE=1 SV=2 - [TPM1_HUMAN] | 851.25 | 50.35 | 2 | 14 | 21 | 40 | 284 | 32.7 | 4.74 |
| P60709 | Actin, cytoplasmic 1 OS=Homo sapiens GN=ACTB PE=1 SV=1 - [ACTB_HUMAN] | 800.30 | 49.33 | 12 | 21 | 21 | 38 | 375 | 41.7 | 5.48 |
| P22626 | Heterogeneous nuclear ribonucleoproteins A2/B1 OS=Homo sapiens GN=HNRNPA2B1 PE=1 SV=2 - [ROA2_HUMAN] | 542.46 | 56.09 | 1 | 19 | 21 | 37 | 353 | 37.4 | 8.95 |
| P40926 | Malate dehydrogenase, mitochondrial OS=Homo sapiens GN=MDH2 PE=1 SV=3 - [MDHM_HUMAN] | 546.71 | 49.70 | 1 | 19 | 19 | 24 | 338 | 35.5 | 8.68 |
| P00558 | Phosphoglycerate kinase 1 OS=Homo sapiens GN=PGK1 PE=1 SV=3 - [PGK1_HUMAN] | 421.57 | 40.29 | 2 | 18 | 18 | 28 | 417 | 44.6 | 8.10 |
| P10412 | Histone H1.4 OS=Homo sapiens GN=HIST1H1E PE=1 SV=2 - [H14_HUMAN] | 866.02 | 40.18 | 4 | 14 | 17 | 31 | 219 | 21.9 | 11.03 |
| Q14847 | LIM and SH3 domain protein 1 OS=Homo sapiens GN=LASP1 PE=1 SV=2 - [LASP1_HUMAN] | 627.48 | 50.96 | 1 | 16 | 16 | 26 | 261 | 29.7 | 7.05 |
| P46777 | 60S ribosomal protein L5 OS=Homo sapiens GN=RPL5 PE=1 SV=3 - [RL5_HUMAN] | 289.55 | 43.10 | 1 | 16 | 16 | 20 | 297 | 34.3 | 9.72 |
| P07195 | L-lactate dehydrogenase B chain OS=Homo sapiens GN=LDHB PE=1 SV=2 - [LDHB_HUMAN] | 563.30 | 40.42 | 3 | 13 | 15 | 27 | 334 | 36.6 | 6.05 |
| P00338 | L-lactate dehydrogenase A chain OS=Homo sapiens GN=LDHA PE=1 SV=2 - [LDHA_HUMAN] | 447.84 | 41.27 | 3 | 13 | 15 | 23 | 332 | 36.7 | 8.27 |
| P09651 | Heterogeneous nuclear ribonucleoprotein A1 OS=Homo sapiens GN=HNRNPA1 PE=1 SV=5 - [ROA1_HUMAN] | 517.23 | 39.52 | 2 | 12 | 14 | 27 | 372 | 38.7 | 9.13 |
| Q14103 | Heterogeneous nuclear ribonucleoprotein D0 OS=Homo sapiens GN=HNRNPD PE=1 SV=1 - [HNRPD_HUMAN] | 326.65 | 33.24 | 1 | 12 | 13 | 16 | 355 | 38.4 | 7.81 |
| P37837 | Transaldolase OS=Homo sapiens GN=TALDO1 PE=1 SV=2 - [TALDO_HUMAN] | 270.08 | 33.83 | 1 | 13 | 13 | 16 | 337 | 37.5 | 6.81 |
| Q96AG4 | Leucine-rich repeat-containing protein 59 OS=Homo sapiens GN=LRRC59 PE=1 SV=1 - [LRC59_HUMAN] | 187.21 | 35.50 | 1 | 13 | 13 | 15 | 307 | 34.9 | 9.57 |
| P16401 | Histone H1.5 OS=Homo sapiens GN=HIST1H1B PE=1 SV=3 - [H15_HUMAN] | 730.38 | 36.28 | 1 | 9 | 12 | 29 | 226 | 22.6 | 10.92 |
| P29692 | Elongation factor 1-delta OS=Homo sapiens GN=EEF1D PE=1 SV=5 - [EF1D_HUMAN] | 723.85 | 46.62 | 1 | 12 | 12 | 28 | 281 | 31.1 | 5.01 |
| P23193 | Transcription elongation factor A protein 1 OS=Homo sapiens GN=TCEA1 PE=1 SV=2 - [TCEA1_HUMAN] | 347.98 | 38.21 | 1 | 12 | 12 | 16 | 301 | 33.9 | 8.38 |
| P30533 | Alpha-2-macroglobulin receptor-associated protein OS=Homo sapiens GN=LRPAP1 PE=1 SV=1 - [AMRP_HUMAN] | 289.80 | 39.78 | 1 | 12 | 12 | 16 | 357 | 41.4 | 8.78 |
| P17174 | Aspartate aminotransferase, cytoplasmic OS=Homo sapiens GN=GOT1 PE=1 SV=3 - [AATC_HUMAN] | 280.53 | 33.41 | 1 | 12 | 12 | 15 | 413 | 46.2 | 7.01 |
| Q01105 | Protein SET OS=Homo sapiens GN=SET PE=1 SV=3 - [SET_HUMAN] | 332.68 | 30.69 | 2 | 11 | 11 | 20 | 290 | 33.5 | 4.32 |
| O75937 | DnaJ homolog subfamily C member 8 OS=Homo sapiens GN=DNAJC8 PE=1 SV=2 - [DNJC8_HUMAN] | 256.28 | 44.27 | 1 | 11 | 11 | 12 | 253 | 29.8 | 9.06 |
| P08670 | Vimentin OS=Homo sapiens GN=VIM PE=1 SV=4 - [VIME_HUMAN] | 222.36 | 22.96 | 3 | 9 | 11 | 13 | 466 | 53.6 | 5.12 |
| P51858 | Hepatoma-derived growth factor OS=Homo sapiens GN=HDGF PE=1 SV=1 - [HDGF_HUMAN] | 145.48 | 51.25 | 1 | 11 | 11 | 16 | 240 | 26.8 | 4.73 |
| Q86YZ3 | Hornerin OS=Homo sapiens GN=HRNR PE=1 SV=2 - [HORN_HUMAN] | 332.59 | 11.33 | 1 | 10 | 10 | 16 | 2850 | 282.2 | 10.04 |
| Q99729 | Heterogeneous nuclear ribonucleoprotein A/B OS=Homo sapiens GN=HNRNPAB PE=1 SV=2 - [ROAA_HUMAN] | 315.40 | 25.60 | 1 | 10 | 10 | 14 | 332 | 36.2 | 8.21 |
| P51991 | Heterogeneous nuclear ribonucleoprotein A3 OS=Homo sapiens GN=HNRNPA3 PE=1 SV=2 - [ROA3_HUMAN] | 247.98 | 20.63 | 1 | 9 | 10 | 14 | 378 | 39.6 | 9.01 |
| Q02878 | 60S ribosomal protein L6 OS=Homo sapiens GN=RPL6 PE=1 SV=3 - [RL6_HUMAN] | 220.29 | 31.60 | 1 | 10 | 10 | 18 | 288 | 32.7 | 10.58 |
| Q9H444 | Charged multivesicular body protein 4b OS=Homo sapiens GN=CHMP4B PE=1 SV=1 - [CHM4B_HUMAN] | 405.13 | 31.25 | 1 | 9 | 9 | 13 | 224 | 24.9 | 4.82 |
| P40121 | Macrophage-capping protein OS=Homo sapiens GN=CAPG PE=1 SV=2 - [CAPG_HUMAN] | 323.07 | 31.90 | 1 | 9 | 9 | 14 | 348 | 38.5 | 6.19 |
| Q13347 | Eukaryotic translation initiation factor 3 subunit I OS=Homo sapiens GN=EIF3I PE=1 SV=1 - [EIF3I_HUMAN] | 260.48 | 37.23 | 1 | 9 | 9 | 12 | 325 | 36.5 | 5.64 |
| P38159 | RNA-binding motif protein, X chromosome OS=Homo sapiens GN=RBMX PE=1 SV=3 - [RBMX_HUMAN] | 194.20 | 27.11 | 2 | 9 | 9 | 11 | 391 | 42.3 | 10.05 |
| P07437 | Tubulin beta chain OS=Homo sapiens GN=TUBB PE=1 SV=2 - [TBB5_HUMAN] | 158.45 | 22.97 | 7 | 3 | 9 | 9 | 444 | 49.6 | 4.89 |
| P08758 | Annexin A5 OS=Homo sapiens GN=ANXA5 PE=1 SV=2 - [ANXA5_HUMAN] | 131.42 | 30.31 | 1 | 9 | 9 | 10 | 320 | 35.9 | 5.05 |
| P68371 | Tubulin beta-4B chain OS=Homo sapiens GN=TUBB4B PE=1 SV=1 - [TBB4B_HUMAN] | 126.81 | 20.45 | 6 | 3 | 9 | 9 | 445 | 49.8 | 4.89 |
| Q9UKM9 | RNA-binding protein Raly OS=Homo sapiens GN=RALY PE=1 SV=1 - [RALY_HUMAN] | 106.25 | 44.44 | 2 | 9 | 9 | 10 | 306 | 32.4 | 9.17 |
| Q9Y3F4 | Serine-threonine kinase receptor-associated protein OS=Homo sapiens GN=STRAP PE=1 SV=1 - [STRAP_HUMAN] | 322.16 | 30.57 | 1 | 8 | 8 | 9 | 350 | 38.4 | 5.12 |
| Q9NZT1 | Calmodulin-like protein 5 OS=Homo sapiens GN=CALML5 PE=1 SV=2 - [CALL5_HUMAN] | 288.51 | 68.49 | 1 | 8 | 8 | 14 | 146 | 15.9 | 4.44 |
| P67936 | Tropomyosin alpha-4 chain OS=Homo sapiens GN=TPM4 PE=1 SV=3 - [TPM4_HUMAN] | 274.63 | 24.60 | 2 | 1 | 8 | 14 | 248 | 28.5 | 4.69 |
| P07910 | Heterogeneous nuclear ribonucleoproteins C1/C2 OS=Homo sapiens GN=HNRNPC PE=1 SV=4 - [HNRPC_HUMAN] | 202.55 | 31.70 | 4 | 8 | 8 | 11 | 306 | 33.6 | 5.08 |
| Q15293 | Reticulocalbin-1 OS=Homo sapiens GN=RCN1 PE=1 SV=1 - [RCN1_HUMAN] | 182.64 | 26.28 | 1 | 8 | 8 | 12 | 331 | 38.9 | 5.00 |
| P40925 | Malate dehydrogenase, cytoplasmic OS=Homo sapiens GN=MDH1 PE=1 SV=4 - [MDHC_HUMAN] | 177.33 | 26.35 | 1 | 8 | 8 | 11 | 334 | 36.4 | 7.36 |
