# Supplementary material for: The Expression of TRIM6 Activates the mTORC1 Pathway by Regulating the Ubiquitination of TSC1-TSC2 to Promote Renal Fibrosis
Source: Front Cell Dev Biol. 2021 Feb 9;8:616747. doi: 10.3389/fcell.2020.616747 (PMC7901959; doi:10.3389/fcell.2020.616747)
Supplement: Supplementary file 2 [file Table_2.DOCX]

**
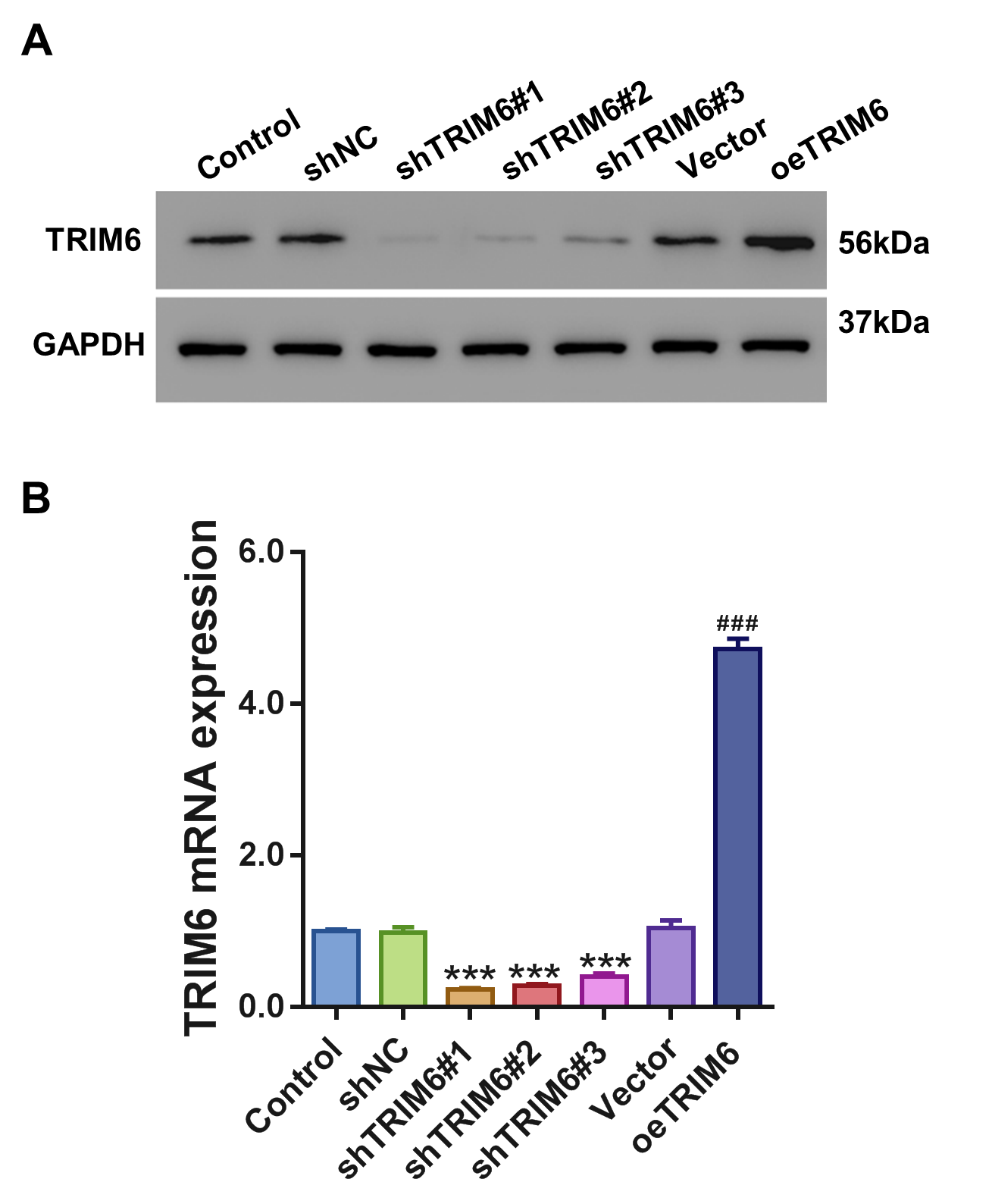
**

**Figure S1. Manipulation of TRIM6 protein expression in HK2 cells.** HK2 cells were transduced with virus expressing *TRIM6* shRNAs (shTRIM6-1#, 2#, 3#) or the control shRNA (shNC), and the recombinant lentivirus (oeTRIM6) or the control vector. The protein expression was examined by western blot (A) and qPCR (B).


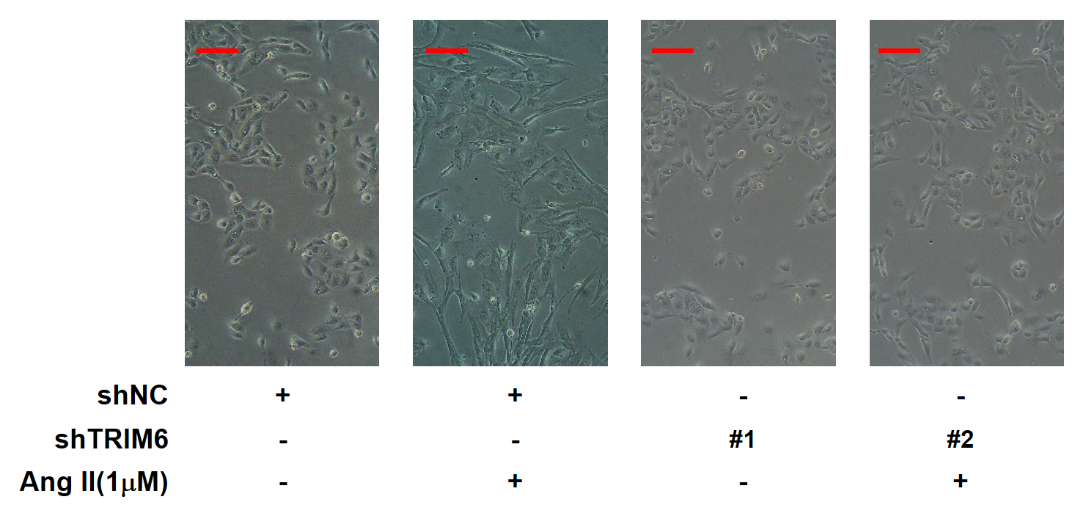


**Figure S2.** Observation of the EMT of HK2 cells with or without the shRNA treatment using phase contrast microscopy. Scale bar: 100 μm.


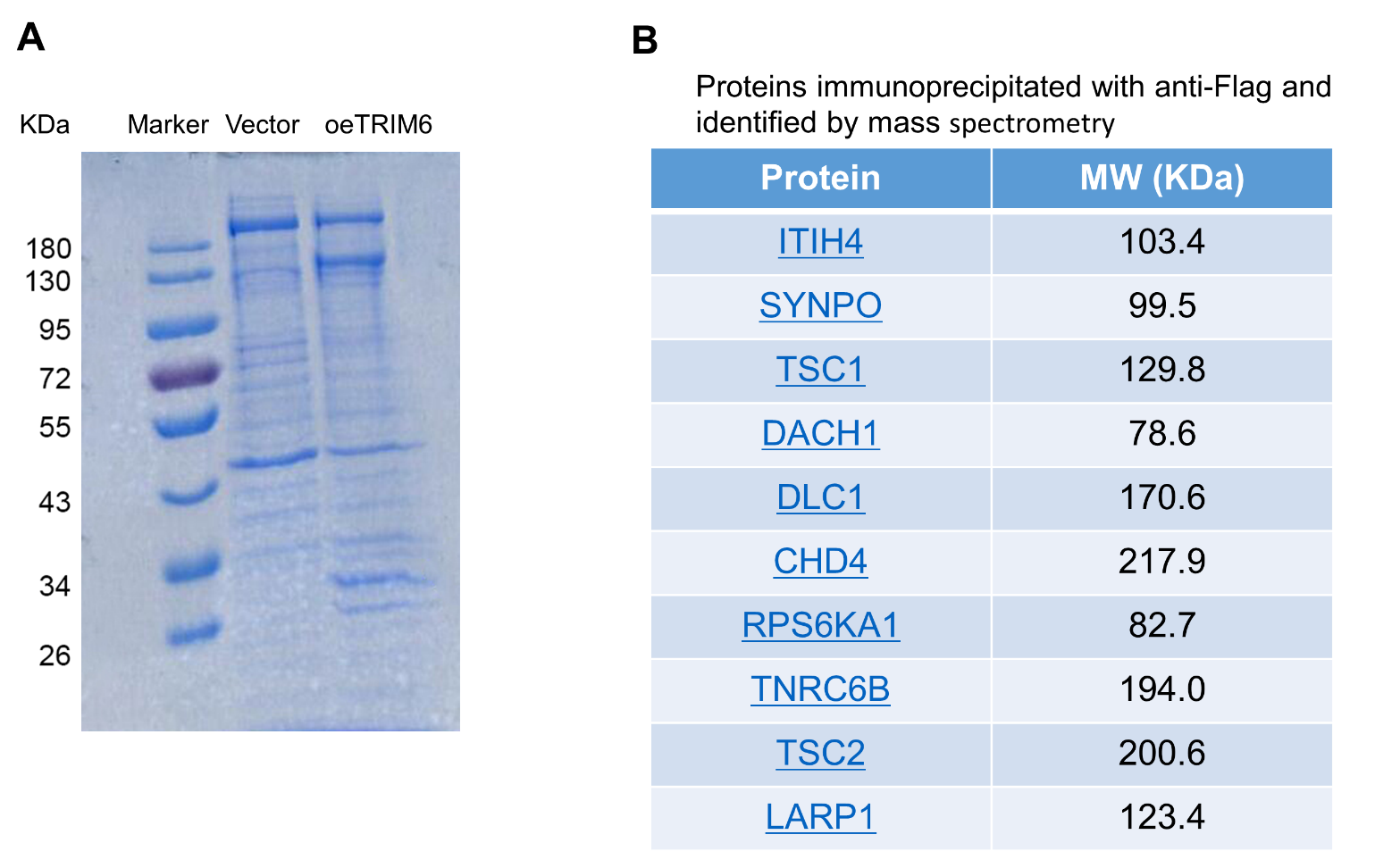


**Figure S3. The results of immunoprecipitation and mass spectrometry to identify TRIM6-associating proteins.** (A) The SDS-PAGE of the proteins immunoprecipitated by TRIM6. (B) The mass spectrometry scores of identified proteins.


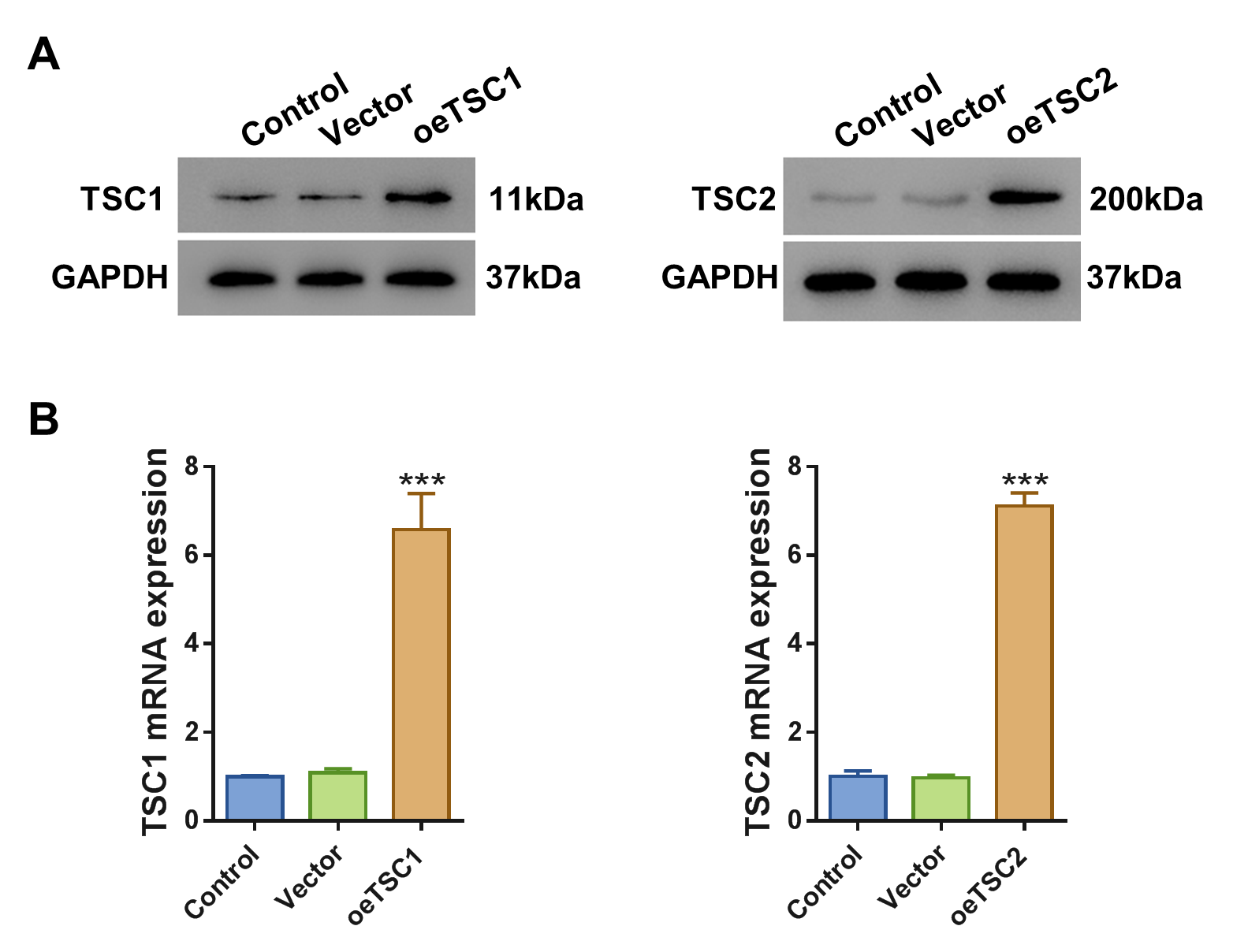


**Figure S4. The overexpression of TSC1 and TSC2 in HK2 cells.** HK2 cells were transduced with the recombinant lentivirus to overexpress TSC1 and TSC2. The expression was examined by western blot (A) and qPCR (B).


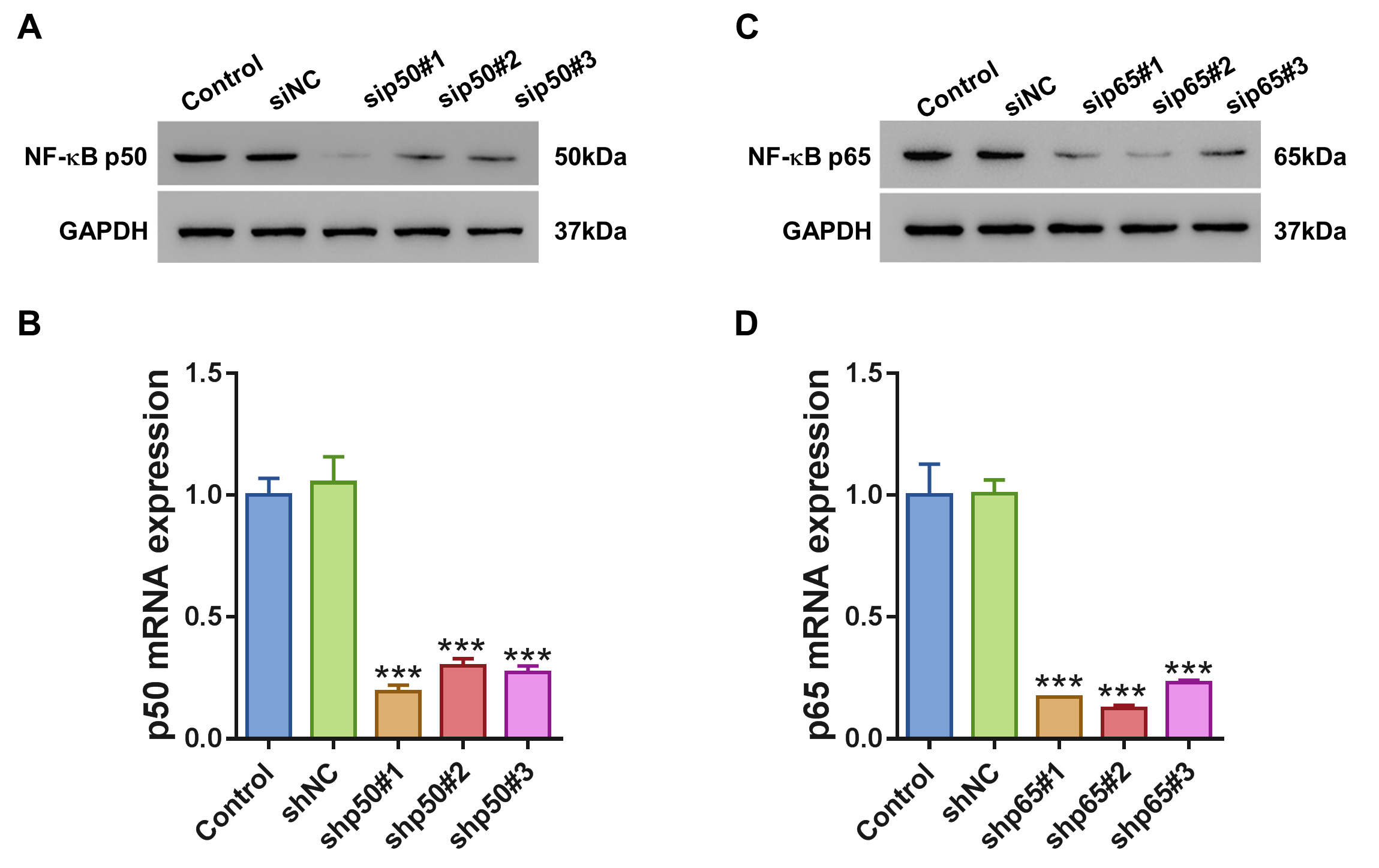


**Figure S5. The knockdown of NF-κB p50 and p65 in HK2 cells.** HK2 cells were transfected with the siRNAs for *NF-κB p65* (sip65-1#, 2#, 3#), *p50* (sip50-1#, 2#, 3#), and the control siRNA (siNC). The effects of RNAi were examined by western blot (A) and (B) and qPCR C) and (D).


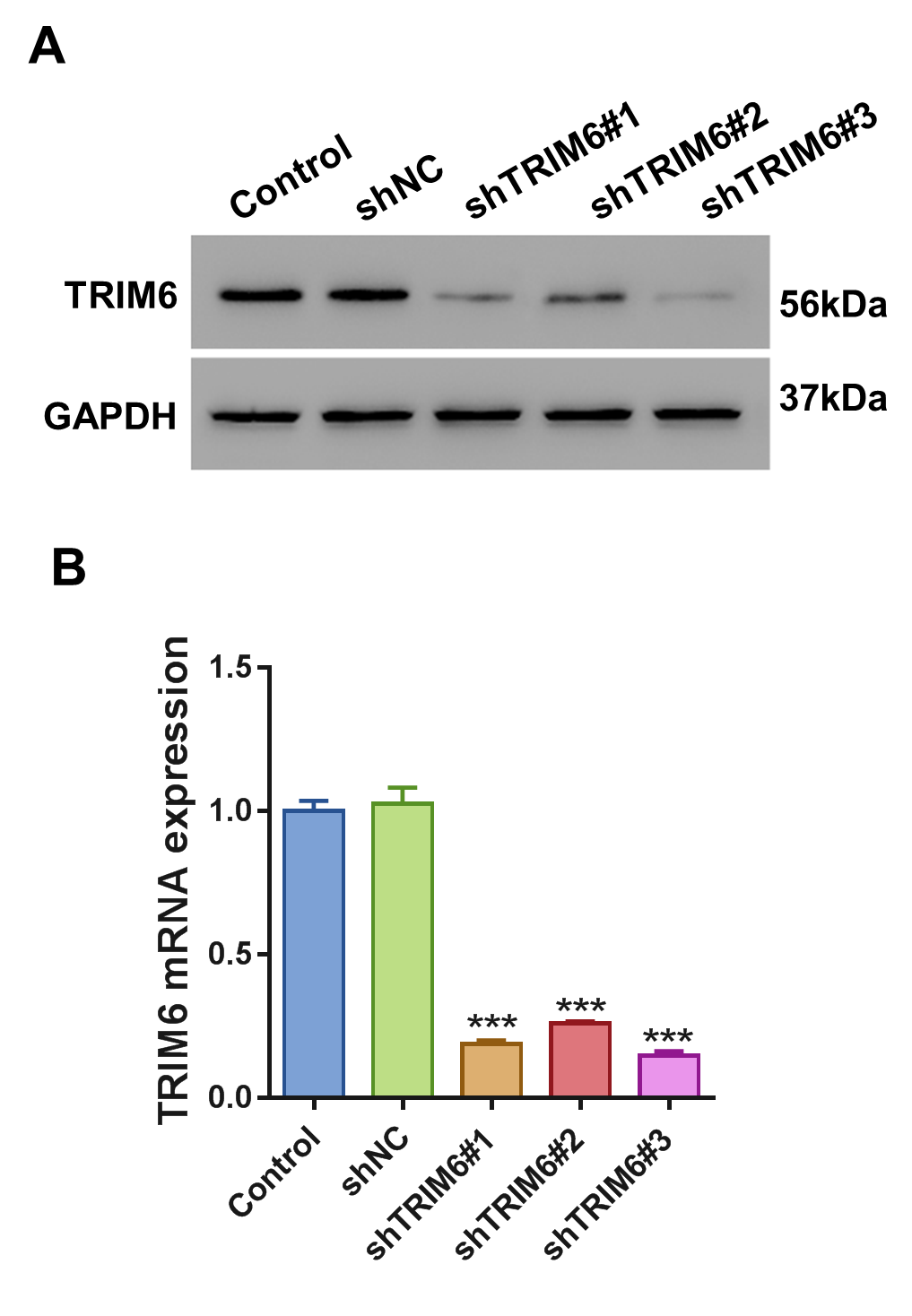


**Figure S6. The knockdown of TRIM6 in rat NRK-52E cells by shRNA.** HK2 cells were transduced with adenovirus expressing *TRIM6* shRNAs (shTRIM6-1#, 2#, 3#) and the control shRNA (shNC). The expression of TRIM6 was examined by western blot (A) and qPCR (B).
